# The Role of NSUN Family Genes in m5C Methylation and Diseases

**DOI:** 10.3390/biomedicines13122951

**Published:** 2025-11-30

**Authors:** Tao Jiang, Nili Jiang, Xuan Chen, Zuming Xiong

**Affiliations:** 1 Department of Radiology, Chongqing University Fuling Hospital, Chongqing University, Chongqing 408000, China; 2 Department of Gastrointestinal Surgery, Chongqing University Fuling Hospital, Chongqing University, Chongqing 408000, China

**Keywords:** NSUN, m5C, cancer, metabolism, inflammatory, diagnosis

## Abstract

5-Methylcytosine (m5C) methylation is a widely present nucleic acid modification in various RNAs and is a reversible epigenetic modification that affects RNA stability, nuclear export, and translation processes. Methylation writers are responsible for adding methyl groups to RNA molecules, regulating gene expression and cellular function through catalyzing methyl transfer reactions. In order to more intuitively demonstrate the important value of NOL1/NOP2/SUN domain (NSUN) family genes in both tumor and non-tumor diseases, we conducted a relevant review. The NSUN family genes (NSUN1/NOP2, NSUN2, NSUN3, NSUN4, NSUN5, NSUN6, NSUN7) are the main writers of m5C methylation. These genes can regulate methylation and affect the expression of other genes and are important in tumor and non-tumor diseases. Pieces of research on 7 NSUN family genes regarding methylation, diagnostic value, inflammatory diseases, cancer, and other diseases were searched for and summarized separately. Differences in NSUN family genes have been observed in many cancers, which can affect tumor growth, metastasis, chemotherapy resistance, and m5C methylation. In addition to affecting cancer, NSUN family genes have also attracted widespread attention due to their involvement in diseases related to growth, development, and metabolism. NSUN2 is the most studied NSUN family gene, which exhibits cancer promoting effects in various cancers such as lung cancer, liver cancer, and colorectal cancer. This review provides an overview of the roles of NSUN family genes in methylation, diagnostic value, inflammatory diseases, cancer, and other diseases.

## 1. Introduction

The NSUN (also known as NOL1/NOP2/SUN domain [1]) family genes are essential m5C modification executors [2]. The NSUN gene family contains seven genes (*NSUN1*, *NSUN2*, *NSUN3*, *NSUN4*, *NSUN5*, *NSUN6*, *NSUN7*). These genes catalyze 5-methylcytosine (m5C) methylation formation at different positions on RNA, which installs the methyl group onto the C5 position of cytosine in RNA [3]. The m5C modification is important in gene expression regulation and cellular function by co-regulating “writers” that add methyl groups, “erasers” that remove methyl groups, and “readers” that recognize methylated RNA. The m5C methylation function includes RNA stability regulation, translation regulation, ribosome biosynthesis, and mRNA fate determination. The process is mediated by methyltransferases, among which the NSUN family is the most important m5C writer. RNA methylation, especially m5C, is an epigenetic modification that is crucial for regulating RNA at the post-transcriptional level. In-depth research on m5C methylation may aid the understanding of disease mechanisms and the development of targeted treatment strategies [4].

NOP2 nucleolar protein (NOP2, also known as NSUN1) [5] is important in accelerating cell proliferation, cell cycle progression, and tumor invasiveness, and is abnormally expressed in cancers [6]. NSUN2 has recently attracted considerable attention due to its crucial role in driving tumor development in an m5C-dependent manner. Xin Yang et al. [7] suggested that m5C formation in mRNA may mainly be catalyzed by NSUN2, and in vitro and in vivo studies have demonstrated that m5C is specifically recognized by the mRNA export adapter ALYREF. NSUN2 regulates nuclear cytoplasmic transport, RNA binding affinity, and related mRNA export of ALYREF [8]. In terms of biochemical reaction, the upregulation of NSUN2 promotes dysregulation of liver glucose and lipid metabolism [9], and promotes inflammation and hyperglycemia during pregnancy [10]. NSUN3 is mainly responsible for specific methylation modifications of mitochondrial tRNA, and mutations in human NSUN3 are associated with mitochondrial diseases [11]. NSUN6 is a methyltransferase that relies on tRNA structure and can independently catalyze m5C formation. NSUN6 has strong recognition activity regarding CUCCA motifs in specific stem loop structures [12].

The study of NSUN family genes has important scientific value and clinical application prospects: (1) disease diagnostic markers, where NSUN family members are abnormally expressed in cancers and can act as potential diagnostic and prognostic markers for tissues and blood; (2) the development of therapeutic targets: the research and development of small molecule inhibitors targeting the NSUN family have progressed; (3) gene therapy applications: reactive oxygen species (ROS)-responsive nanoparticles delivering small interfering RNA (siRNA) targeting NSUN family genes have yielded promising results in some cancer treatments, presenting new avenues for targeted therapy of RNA methylation related diseases; and (4) metabolic regulation research: research on NSUN family genes has continued to heat up, especially in the field of oncology. In the present review, we collected and analyzed experimentally validated data and articles to clarify the role of NSUN family genes in human diseases to provide new insights for the translation of experimental results into clinical practice.

## 2. Role in Methylation

### 2.1. NOP2 (NSUN1) in Methylation

NOP2 is an S-adenosyl-L-methionine-dependent methyltransferase that regulates cell cycle and proliferation activity. NOP2 regulates c-Myc expression through m5C modification to promote glycolysis. Previous studies have indicated that m5C methylation induces *c-Myc* mRNA degradation in an EIF3A-dependent manner. NOP2 increased the expression of the glycolytic genes *LDHA*, *TPI1*, *PKM2*, and *ENO1* [13]. RNA sequencing performed to identify downstream targets of *NOP2* demonstrated that overexpressing *NOP2* significantly upregulated *EZH2* mRNA expression. Subsequent validation experiments have demonstrated that the effect of NOP2 on *EZH2* mRNA stability depends on m5C. Furthermore, NOP2 and the m5C recognition protein ALYREF have a co-regulatory relationship, where ALYREF regulates *EZH2* mRNA stability [14]. NOP2 stimulates m5C modification of *APOL1* mRNA, and the m5C recognition protein YBX1 stabilizes *APOL1* mRNA by recognizing and binding to the m5C site in the 3′-untranslated region (3′UTR) [15]. NOP2 binds with HIV-1 TAR RNA at the 5′-long terminal repeat and leads to its m5C methylation [16].

NOP2 overexpression enhanced XPD expression by elevating the m5C methylation of XPD, which contributed to inhibiting the proliferation, migration, and invasion of hepatocellular carcinoma (HCC) cells. In summary, the study demonstrated that NOP2-mediated m5C methylation modification increased *XPD* mRNA stability and inhibited HCC malignant progression [17]. In colorectal cancer (CRC), NOP2 promotes m5C methylation of *LMNB2* mRNA and enhances its stability, increasing LMNB2 protein levels [18]. The regulatory mechanism of NOP2 on RAPGEF4 in ovarian cancer depends on the m5C methylation level. Reducing NOP2 expression in ovarian cancer decreases m5C levels, while increasing NOP2 expression increases m5C levels. Lastly, knocking down NOP2 in ovarian cancer reduced the m5C methylation level of *RAPGEF4* mRNA [5].

### 2.2. NSUN2 in Methylation

NSUN2 enhances the m5C methylation stability of *PTPRD* mRNA, exacerbating A1 astrocyte activation and the subsequent inflammation-mediated tissue damage after traumatic brain injury [19]. In retinoblastoma studies, NSUN2 and YBX1 mediated the m5C modification and mRNA stability of *HKDC1* [20], where the global and mRNA m5C levels of retinoblastoma were significantly higher than those of normal retina. Mechanistically, NSUN2 recognizes m5C modifications of PFAS through ALYREF, enhancing the RNA stability of *PFAS* to increase its expression [21].

RNA m5C hypermethylation and NSUN2 were significantly correlated with intrinsic resistance to EGFR-TKIs. NSUN2 methylated the QSOX1 coding sequence (CDS) region, enhancing *QSOX1* translation through m5C recognition of protein YBX1 [22]. Methylated- RNA immunoprecipitation (MeRIP)-qPCR assay results demonstrated that the m5C level of *NRF2* mRNA was higher when NSUN2 was overexpressed in non-small-cell lung cancer (NSCLC) cells. Mechanistically, NSUN2 upregulates NRF2 expression by enhancing *NRF2* mRNA stability, which is recognized by YBX1 through m5C modifications within its 5′UTR region, rather than affecting its translation [23]. Knocking down NSUN2 in A549 and SPAC-1 cells decreased the global m5C levels. PIK3R2 was upregulated by NSUN2-mediated m5C methylation by enhancing its mRNA stabilization [24]. In lung cancer, NSUN2-mediated m5C modification promoted mRNA stability of *ME1*, *GLUT3*, and *CDK2*, leading to metabolic reprogramming and cell cycle changes [25].

The mRNA m5C methylation level was significantly upregulated in esophageal squamous cell carcinoma (ESCC) tissues. Knocking out NSUN2 in the ESCC cells significantly reduced the m5C methylation level, which was increased in NSUN2-overexpressing ESCC cells [26,27]. These results indicated that NSUN2 is crucial in YBX1-mediated ESCC progression. Furthermore, knocking down NSUN2 significantly reduced the m5C level of SMOX in KYSE150 cells, and the SMOX CDS contains an m5C site, which is highly consistent with the YBX1 binding site. In addition, RT-qPCR and Western blot analysis determined that ectopic expression of YBX1 or NSUN2 significantly increased the SMOX expression level. Notably, wild-type (WT) YBX1/NSUN2, instead of their mutants, could upregulate SMOX protein expression in ESCC cells. These results strongly indicated that the sustained expression of SMOX depends on the m5C catalytic activity of NSUN2 and the m5C binding ability of YBX1 [26]. Mechanistically, NSUN2 induces m5C modification of GRB2 and stabilizes its mRNA, which is mediated by the novel m5C mediator LIN28B [27].

In the mechanism of anaplastic thyroid cancer (ATC), NSUN2 catalyzes m5C modification related to tRNA structure, stabilizing tRNA to maintain homeostasis and rapidly transporting amino acids, especially leucine. This stable tRNA significantly improves the efficiency required to support cancer-promoting translation programs, including c-Myc, BCL2, RAB31, JUNB, and TRAF2 [28]. In ATC, NSUN2 acts as the m5C writer, while ALYREF acts as the reader of *SRSF6* mRNA. Under NSUN2 regulation, ALYREF transports m5C-modified *SRSF6* mRNA from the nucleus to the cytoplasm [29].

NSUN2 stabilizes *FABP5* mRNA by inducing m5C modification, further promoting fatty acid metabolism in osteosarcoma cells [30]. RIP-seq (RNA immunoprecipitation-sequencing) and MeRIP-seq (methylated- RNA immunoprecipitation-sequencing) identified SARS2 as a downstream target of NSUN2. Silencing NSUN2 in HCC Huh7 cells downregulated SARS2 expression and m5C methylation [31]. MeRIP confirmed that NSUN2 promotes m5C modification of SOAT2, and the m5C level was elevated in HCC tissue samples [32]. GPX4 is the main inhibitor of liver cell ferroptosis, and downregulating NSUN2 reduced the m5C methylation of the *GPX4* mRNA 3′UTR [33].

In gastric cancer (GC), methylation sequencing and RT-qPCR confirmed that knocking out NSUN2 significantly decreased PIK3R1 and PCYT1A expression levels. Therefore, *PIK3R1* and *PCYT1A* may be target genes of NSUN2-modified m5C [34]. In CRC, NSUN2 induces m5C modification of SKIL and stabilizes its mRNA, which is mediated by YBX1 [35]. In terms of the molecular mechanism, downregulating NSUN2 decreased in m5C methylation in CRC cells, and decreased *SLC7A11* mRNA translation and stability [36]. NSUN2 and YBX1 were identified as the m5C methylation writer and reader, respectively, of ENO1 in the reprogramming of glucose metabolism and lactate mechanisms in CRC [37]. In the mechanism of colitis, RoRγt recruits NSUN2 to the chromatin regions of its target genes, including *IL17A* and *IL17F*, leading to the formation of transcription-related m5C and enhancing mRNA stability [38].

ALYREF recognizes hypermethylated m5C site of NSUN2, resulting in NSUN2 upregulation in urothelial carcinoma of the bladder (UCB) [39]. NSUN2 has been identified as a regulatory factor of R-loop, which can bind and stabilize the R-loop structure through a process dependent on its m5C catalytic activity [40]. NSUN2 enhances m5C modification of mRNA, promoting endometrial cancer cell proliferation. Mechanistically, NSUN2 stimulates m5C modification of *SLC7A11* mRNA, while YBX1 directly recognizes and binds to the m5C site on *SLC7A11* mRNA through its internal cold shock domain, increasing stability and the levels of *SLC7A11* mRNA [41].

In acute myeloid leukemia (AML), NSUN2 stabilizes *PHGDH* and *SHMT2* mRNA and upregulates their expression by regulating m5C modification [42]. In the AML mechanism, NSUN2 catalyzes m5C deposition on the 3′UTR of *FSP1* mRNA, promoting its recognition and stabilization by the m5C recognition protein YBX1 [43]. In human cortical epithelial cells (HCEC), silencing NSUN2 almost did not alter *UHRF1* mRNA expression levels, although it reduced UHRF1 protein expression levels, supporting NSUN2 regulation of *UHRF1* translation. These data indicated that UHRF1 is a direct target of NSUN2, where NSUN2 mainly promotes *UHRF1* translation by increasing ALYREF [44].

Uveal melanoma (UVM) cells and tissues demonstrated significantly elevated global RNA m5C levels, which were significantly decreased by downregulating NSUN2. Bioinformatic analyses, m5C-RIP-qPCR, and luciferase assay identified CTNNB1 as a direct target of NSUN2-mediated m5C modification in UVM cells. CTNNB1 protein levels were downregulated in NSUN2 knockdown UVM cells, while *CTNNB1* mRNA levels remained unchanged. NSUN2-mediated CTNNB1 methylation status regulated UVM cell proliferation and migration. Overexpressing miR-124a in UVM cells reduced the NSUN2 expression level, indicating that it is an upstream regulatory factor of this response [45]. In diffuse large B-cell lymphoma (DLBCL) cells, NSUN2 stabilized *PDL1* mRNA through an m5C-dependent mechanism and YBX1-dependent pathway. Moreover, NSUN2 in extracellular vesicles derived from DLBCL cells stabilized PDL1 in a YBX1-dependent manner [46].

In 3T3-L1 cells, NSUN2 directly targeted the mRNA of the key inhibitory regulator of cell cycle progression (*CDKN1A*), and upregulated its protein expression in an m5C-dependent manner. ALYREF recognition of *CDKN1A* mRNA was inhibited in the absence of NSUN2, decreasing the transport of *CDKN1A* mRNA from the nucleus to the cytoplasm [47]. In cervical cancer, NSUN2 promotes m5C modification on KRT13, and m5C-methylated KRT13 is recognized and stabilized by the m5C reader protein YBX1 [48]. Upregulating ICAM-1 in vascular endothelial inflammation requires NSUN2. NSUN2 methylation promoted ICAM-1 translation, increasing the adhesion between white blood cells and endothelial cells [49].

The elevation of plasma homocysteine (Hcy, 100 µM) upregulated *Nsun2* expression in rat T lymphocyte tRNA, where *Nsun2* methylated *IL-17A* mRNA in m5C mode. The methylation of NSun2-methylated *IL-17A* mRNA at cytochrome C466 in vitro and vivo promoted the translation of IL-17A. NSun2 mediated hyperhomocysteinemia-induced upregulation of IL-17A expression by methylating *IL-17A* mRNA and promoting its translation in T lymphocytes [50]. In a study of the mechanism of 293T cells and type 2 diabetes mellitus, NSUN2 deficiency reduced ACSL6 expression by inhibiting m5C modification of *ACSL6* mRNA [9].

In tumors, NSUN2 activation maintains global m5C RNA methylation, including that of TREX2, and stabilizes TREX2 [51]. After enhancing NSUN2 activity, it targeted *GCLC* mRNA to promote m5C formation and *GCLC* mRNA stability [52]. NSUN2 specifically mediated m5C methylation of *IRF3* mRNA and accelerated its degradation, decreasing IRF3 and downstream IFN-β levels [53]. In cardiomyocytes, NSUN2 significantly promoted PRKACA translation by YBX1-specific methylation of *PRKACA* mRNA [54]. During fetal development, NSUN2 recruited the JARID2–EZH2 complex and regulated its activity through m5C-dependent recognition by ALYREF [55].

### 2.3. NSUN3 in Methylation

PD-L1 levels were downregulated following NSUN3 downregulation in A549 and PC9 cells. Additionally, inhibiting NSUN3 promoted *PDL1* mRNA degradation. A series of experiments demonstrated that NSUN3 stabilized *PDL1* mRNA in an m5C-dependent manner [56]. In the mechanism of sepsis-associated acute kidney injury, NSUN3 increased the stability of *TIFA* mRNA and upregulated its expression through m5C modification. Eventually, NSUN3 enhanced sepsis-associated acute kidney injury by stabilizing *TIFA* mRNA through m5C [57]. NSUN3-mediated m5C modification enhanced *TAK1* mRNA stability in HULEC-5a cells during sepsis-induced pulmonary injury. Lastly, NSUN3-mediated m5C modification promoted sepsis-induced pulmonary injury by regulating inflammation [58].

### 2.4. NSUN4 in Methylation

In the mechanism of NSCLC, NSUN4 enhances *CDC20* mRNA stability through m5C modification [59]. In the glioma mechanism, NSUN4-mediated m5C changes regulate ALYREF binding to *CDC42* mRNA, affecting *CDC42* mRNA stability [60]. NSUN4 can increase m5C methylation in lung cancer cells. NSUN4 can act as an m5C writer, mediating the m5C modification of circERI3. Silencing NSUN4 reduced the cytoplasmic distribution of circERI3, while overexpressing NSUN4 increased the nuclear export of circERI3, which increased cytoplasmic distribution [61]. In cartilage differentiation, NSUN4 and METTL3 jointly regulated m5C and m6A methylation of *SOX9* by binding to its 3′UTR [62].

### 2.5. NSUN5 in Methylation

In gliomas, NSUN5 directly interacts with CTNNB1 chromium-associated RNA (caRNA) and deposits m5C on it. The recruitment of TET2 to chromatin induced by NSUN5 is not related to its methyltransferase activity. Additionally, NSUN5 enhanced chromatin recruitment of RBFOX2, which acted as a 5hmC-specific recognition factor to recognize and promote the degradation of 5hmC caRNA [63]. NSUN5 methylates cytosine 3782 of 28S rRNA in glioblastoma cells [64]. In esophageal cancer, NSUN5 is associated with METTL1 and positively regulates its expression, where NSUN5 directly binds to the METTL1 transcript, promoting its m5C modification in esophageal cancer cells [65].

MeRIP-seq and RIP-seq demonstrated that ZBED3 acts as a new downstream target of NSUN5 in HCC cells. Knocking down NSUN5 significantly reduced the m5C modification and expression of ZBED3. Consequently, NSUN5 regulated its target gene *ZBED3* through m5C modification to promote HCC development [66]. Furthermore, NSUN5 stabilized EFNA3 in HCC by promoting m5C modification of EFNA3 [67]. In cholangiocarcinoma, NSUN5-mediated m5C modification is located at the 137 C site of the glutaminase mRNA sequence, stabilizing glutaminase mRNA and leading to the accumulation of intracellular glutaminase [68]. In CRC, knocking down NSUN5 significantly reduced the mRNA expression and half-life of GPX4 [69].

In clear cell renal cell cancer (ccRCC), upregulating NSUN5 mediated m5C modification of mRNA in ccRCC cells to promote *ENO3* mRNA stability and expression [70]. In the mechanism of prostate cancer, CDK13 interacts with NSUN5, promoting its phosphorylation at the Ser327 site. Conversely, phosphorylated NSUN5 catalyzes m5C modification of *ACC1* mRNA, which binds to ALYREF to enhance its stability and nuclear output, promoting ACC1 expression and lipid deposition in prostate cancer cells [71].

Dot blot assays demonstrated that overall RNA m5C levels decreased during ferroptosis. NSUN5 binding to *SLC7A11* mRNA enhanced its protein translation and conferred it with the ability to resist ferroptosis [72]. In RAW264.7 cells, NSUN5 interacted with CX3CL1 and inhibited its stability by promoting m5C modification of *CX3CL1* mRNA [73]. The absence of NSUN2 in HeLa cells affected both UPF1 steady-state levels and binding to target mRNAs [74]. In Nsun5 knockout (Nsun5-KO) mice, knocking out Nsun5 decreased the m5C level, where Nsun5 deficiency influenced MAD2L2 and GDF9 translation efficiency in the ovary [75].

### 2.6. NSUN6 in Methylation

NSUN6 is a novel mammalian m5C tRNA methyltransferase. In ESCC, NSUN6-mediated tRNA m5C modification selectively enhances the translation efficiency of *CDH1* mRNA, which is codon-dependent [76]. In lung cancer cells, NSUN6 regulates NM23-H1 expression through m5C modification of the *NM23-H1* mRNA 3′UTR [77]. In HCC cells, BMPER is a downstream target of NSUN6, which can stabilize BMPER expression in an m5C-dependent manner [78]. NSUN6 upregulates the expression of the oncogene METTL3 and catalyzes its m5C modification in colon cancer cells [79].

The tRNA(Cys) and tRNA(Thr) are RNA substrates of NSUN6, and the cytosine C72 at the 3′ end of the tRNA acceptor stem has been identified as the target nucleoside [80,81]. NSUN6 methylation of tRNA occurs in late-stage tRNA biosynthesis after the addition of the CCA tail and export of the tRNA from the nucleus. NSUN6-mediated methylation requires the presence of a 3′-CCA in the tRNA substrates [81].

Bioinformatics analysis and RIP and MeRIP assays identified and validated EEF1A2 as a potential target of NSUN6 in osteosarcoma. Knocking down NSUN6 significantly inhibited EEF1A2 expression, as the stability of *EEF1A2* mRNA decreased in an m5C-dependent manner [82]. In osteosarcoma, decreased NSUN6 expression reduced m5C modifications on *PEX1* and *PEX3* mRNA, leading to PEX1 and PEX3 instability by losing the binding of the m5C recognition protein YBX1 [83].

The integration of m5C-seq, mRNA-seq, and functional validation identified *NDRG1* as a downstream target gene of NSUN6 in cervical cancer. NSUN6 promoted m5C modification of *NDRG1* mRNA, while ALYREF bound to m5C-labeled *NDRG1* mRNA and enhanced its stability [84]. Lastly, NSUN6 promoted HDAC10 expression by mediating m5C methylation and inhibited the transcription of chemotactic factors associated with macrophages [85].

### 2.7. NSUN7 in Methylation

NSUN7 epigenetic inactivation is common in HCC cells. The epigenetic deletion of NSUN7 induced mRNA hypomethylation and protein downregulation of the RNA-binding protein CCDC9B. Furthermore, NSUN7 epigenetic loss identified primary HCC with shorter overall survival (OS), where epigenetically inactivated NSUN7 in HCC prevented proper mRNA methylation [86]. Silencing NSUN7 reduces pyroptosis of granulosa cells by inhibiting m5C methylation of NLRP3, thereby slowing down the progression of polycystic ovary syndrome [87].

Finally, Table 1 summarizes the methylation relationship between NSUN family genes and other genes.

## 3. Role in Body Fluid Biomarking

NOP2 and NSUN7 were upregulated in the peripheral blood mononuclear cells (PBMCs) of sepsis patients [areas under the ROC curve (AUC) = 0.707 and 0.828, respectively], indicating that NSUN7 may act as new potential diagnostic biomarkers and therapeutic targets [88]. The *NOP2* rs3764909 and *NSUN4* rs10252 variants enhanced the risk of acute lymphoblastic leukemia in children and are considered potential biomarkers of pediatric acute lymphoblastic leukemia [89].

NSUN2, NSUN6, and NSUN7 were highly expressed in the blood and kidney of diabetic nephropathy mice [90]. Summary data-based Mendelian randomization and meta-analysis results confirmed that NSUN4 is a key regulatory factor of natural age at menopause in terms of gene expression and DNA methylation levels. Animal experiments have demonstrated that NSUN4 levels in the blood reflected decreased ovarian function, suggesting that NUSN4 levels are a potential biomarker of ovarian aging [91]. Plasma exosomes were isolated from myeloperoxidase-antibody-associated vasculitis (MPO-AAV) patients and healthy controls. The subsequent sequencing and RT-qPCR demonstrated that the MPO-AAV patients had significantly downregulated extracellular vesicle NSUN4 compared with the healthy controls [92]. A survey of 402 patients with neuroblastoma and 473 controls, followed by TaqMan analysis, revealed that *NSUN4* rs10736428 polymorphism and increased susceptibility to neuroblastoma were closely associated [93].

The blood immune cells of patients with CRC had significantly increased m5C RNA content. The m5C levels increased with CRC progression and metastasis, but decreased after treatment. Hence, the increased NSUN5 expression in the blood of patients with CRC may be a promising non-invasive diagnostic biomarker of CRC [94]. RNA-Seq and RT-qPCR validation of peripheral blood samples revealed that *NSUN6* is a key gene in rheumatoid arthritis-associated interstitial lung disease, where its expression was significantly lower than that of rheumatoid arthritis-only patients [95].

## 4. Role in Inflammatory Diseases

### 4.1. NOP2 (NSUN1) in Inflammatory Diseases

NOP2 is a limiting factor of HIV-1, and a loss- and gain-of-function analysis confirmed that NOP2 restricts HIV-1 replication. Reducing NOP2 expression promoted the reactivation of latent HIV-1 pre-viruses in various cell lines and primary CD4+ T cells, whether alone or in combination with latent reversal agents. Hence, the study verified that NOP2 inhibits HIV-1 transcription and promotes viral latency [16].

### 4.2. NSUN2 in Inflammatory Diseases

NSUN2 deficiency negatively regulated hepatitis B virus (HBV) expression, while TET2 deficiency positively regulated it. Compared with WT HepG2-NTCP cells and primary human liver cells, cells in which NSUN2 had been knocked down had decreased HBV replication level, and the infection and replication abilities of the mutant virus were significantly impaired in the cells. The expression of HBV and core proteins promoted NSUN2 endogenous expression. Subsequently, NSUN2-mediated m5C modification promoted HBV RNA stability [96].

Compared with the normal control group, NSUN2 expression was significantly increased in rheumatoid arthritis patients and collagen-induced arthritis rats. Knocking down NSUN2 blocked the Wnt–β-catenin signaling pathway and inhibited pathological factors such as MMP3, fibronectin, and interleukins in rheumatoid arthritis. Further experimental verification demonstrated that FTO inhibited rheumatoid arthritis through the NSUN2–SFRP1–Wnt–β-catenin signaling axis [97].

Ischemia–reperfusion injury significantly increased nerve defects, m5C levels, and NSUN2 expression, where knocking out NSUN2 reduced brain damage. Histone lactylation transcriptional activation of NSUN2 promoted m5C-dependent astrocyte neuroinflammation and exacerbated cerebral ischemia–reperfusion injury [98]. Hence, NSUN2 expression and m5C levels were significantly increased in the traumatic brain injury model, with strong colocalization with glial fibrillary acidic protein. Knocking down NSUN2 significantly reduced traumatic brain injury-induced brain damage, inhibited A1 astrocyte activation, reduced the release of pro-inflammatory cytokines, and decreased the proportion of GFAP+C3+/GFAP+S100A10+, while overexpressing PTPRD reversed the inhibitory effect of NSUN2 knockdown on A1 phenotype activation. In vivo validation demonstrated that NSUN2 promoted A1 astrocyte activation through PTPRD, exacerbating traumatic brain injury-induced brain damage [19].

Th17 is a CD4+ T helper cell subset that participates in the inflammatory response in the autoimmune system. The absence of Nsun2 in mouse CD4+ T cells specifically inhibited Th17 cell differentiation and alleviated colitis caused by Th17 cells [38]. Knocking out or knocking down NSUN2 in vitro enhanced type I interferon and downstream ISGs in various viral infections. NSUN2 decreased the levels of IRF3 and downstream IFN-β. In vivo, Nsun2+/− mice had a significantly enhanced innate antiviral response compared to Nsun2+/+ mice. Infection with Sendai virus, vesicular stomatitis virus, herpes simplex virus 1, Zika virus, or SARS-CoV-2 reduced endogenous NSUN2 levels. Following this decrease, the antiviral response could be enhanced and the virus could be effectively cleared [53].

### 4.3. NSUN3 in Inflammatory Diseases

NSUN3 and TIFA expressions were upregulated in mice with sepsis-associated acute kidney injury and lipopolysaccharide (LPS)-induced HK-2 cells. Knocking down NSUN3 alleviated LPS induced HK-2 cell damage and sepsis-associated acute kidney injury in mice by reducing TIFA expression [57]. NSUN3 expression was upregulated in CLP-induced rat lung tissue and LPS-stimulated HULEC-5a cells. Inhibiting NSUN3 alleviated lung injury in the aforementioned cell and animal models, and reduced the levels of inflammatory cytokines. NSUN3 enhanced TAK1 expression, while TAK1 overexpression counteracted the anti-inflammatory effect caused by NSUN3 knockdown. NSUN3-mediated TAK1 m5C modification promoted sepsis-induced pulmonary injury by regulating inflammation [58].

### 4.4. NSUN5 in Inflammatory Diseases

RT-qPCR verified that pulmonary fibrosis tissue samples contained a significantly increased level of NSUN6 expression compared with normal mouse lung tissue samples, while the level of NSUN5 expression was lower [99]. NSUN5 enhanced the inner signaling pathway of RIG-I. NSUN5 deficiency enhanced RNA virus proliferation and inhibited the induction of downstream antiviral genes. Consequently, mice lacking NSUN5 were more susceptible to RNA virus infection than their WT littermates. Mechanistically, NSUN5 directly binds to viral RNA and RIG-I, synergistically enhancing RIG-I recognition of double-stranded RNA [100].

### 4.5. NSUN6 in Inflammatory Diseases

Chronic intermittent hypoxia increased the level of NSUN6 in adipose tissue, leading to in vitro ferroptosis and polarization of M1 macrophages, thereby triggering an inflammatory response. Inhibiting NSUN6 in macrophages prevented chronic intermittent hypoxia-induced oxidative stress and the inflammatory response in adipose tissue [101].

**Figure 1:**
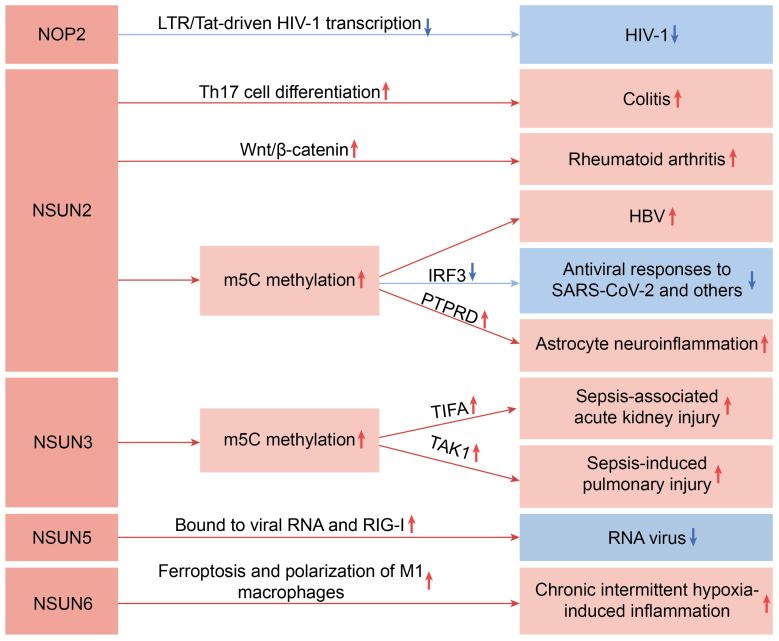
Schematic diagram of the main pathways through which the NSUN family genes influence inflammatory diseases. The red upward arrow represents an increase, and the blue downward arrow represents a decrease.

Figure 1 illustrates the main pathways through which the NSUN family genes influence inflammatory diseases.

## 5. Role in Cancer

### 5.1. NOP2 (NSUN1) in Cancer

NOP2 expression is significantly upregulated in lung adenocarcinoma (LUAD) tissues and cells. In vivo experiments have demonstrated its ability to promote the growth and metastasis of xenograft tumors. The NOP2–ALYREF–EZH2 axis promotes lung cancer [14]. Research results have indicated that A549 cells had significantly higher *NOP2* mRNA and protein expression than Beas-2b cells. In LUAD, elevated NOP2 levels were associated with decreased OS and advanced clinical staging. In vitro experiments have demonstrated that downregulating NOP2 reduced A549 cell proliferation, migration, and invasion. Additionally, NOP2 regulated caspase-3-mediated apoptosis [102].

NOP2 is highly expressed in HCC and is associated with poor prognosis, where it promotes HCC progression by promoting aerobic glycolysis. Combining NOP2 knockout and sorafenib enhanced sensitivity to sorafenib, significantly inhibiting tumor growth. Notably, adenovirus-mediated NOP2 knockout maximized the anti-tumor effect and prolonged the survival of patient-derived tumor xenograft (PDX) mice [13]. NOP2 and XPD were downregulated in HCC tissues and cells. In vitro experiments have demonstrated that overexpressing NOP2 enhanced XPD expression by increasing m5C methylation, thereby inhibiting HCC cell proliferation, migration, and invasion. In summary, that study suggested that NOP2-mediated m5C methylation modification enhanced *XPD* mRNA stability, inhibiting the malignant progression of HCC [17].

NOP2 expression is elevated in GC tissue, where high NOP2 expression is significantly correlated with tumor size, invasion depth, and lymph node metastasis. Additionally, patients with high expression of NOP2 have poor OS, and NOP2 has been identified as an independent prognostic factor. A study that used GC cells determined that NOP2 promoted GC cell proliferation in vitro and in vivo [103]. Compared with the healthy control group, NOP2 expression was significantly upregulated in colon cancer tissues and cells, while the NOP2 knockdown group demonstrated significantly inhibited colon cancer cell proliferation, migration, and invasion [104]. Other studies have reported that NOP2 promotes CRC progression by upregulating m5C methylation of LMNB2 [18].

NOP2 expression is also significantly upregulated in ccRCC tissues and associated with a poor prognosis. *NOP2* is an oncogene in ccRCC that promotes proliferation, migration, and invasion, relying on m5C to stabilize APOL1 and regulate the PI3K–AKT signaling pathway by promoting tumor progression [15]. *NOP2*, *NSUN2*, and *NSUN5* mRNA were significantly upregulated in renal cell carcinoma cell lines (786-O, Caki-1) compared to a human tubular epithelial immortalized cell line (HK-2), while NSUN4 was downregulated in the renal carcinoma cell lines. Furthermore, *NOP2*, *NSUN2*, and *NSUN5* mRNA were significantly upregulated in renal cell carcinoma tissue, while *NSUN4* mRNA was downregulated. The renal cancer cell lines (786-O, Caki-1) had a significantly higher m5C RNA modification level than the HK-2 cell line [105]. LINC00963 facilitated NOP2 by sponging the cancer inhibitor miR-542-3p to promote prostate cancer metastasis, where NOP2 promoted prostate cancer invasion by activating the epithelial–mesenchymal transition (EMT) pathway [106].

NOP2 is significantly upregulated in high-grade serous ovarian cancer tissues. Experimental research has demonstrated that NOP2 promoted cell proliferation in vivo and in vitro through RAPGEF4, and enhanced the migration and invasion ability of high-grade serous ovarian cancer cells in vitro. The regulation of NOP2 and RAPGEF4 may depend on the level of m5C methylation [5].

### 5.2. NSUN2 in Cancer

NSUN2 expression is elevated in ESCC tissues, and NSUN2 promoted ESCC cell proliferation, migration, and invasion in vitro. Hematoxylin–eosin staining demonstrated that knocking down NSUN2 significantly reduced the lung metastasis colonization ability of ESCC cells [26]. E2F1 regulated the abnormal expression of NSUN2 positively. High NSUN2 levels indicated poor survival rates in patients with ESCC. The results indicated that NSUN2 enhances ESCC initiation and progression through m5C-LIN28B-dependent *GRB2* transcript stability. The elevated level of GRB2 increased the activation of the PI3K–AKT and ERK–MAPK signaling pathways [27].

NSUN2 was upregulated in nasopharyngeal carcinoma and predicted poor prognosis in a Gene Expression Omnibus (GEO) dataset and tissue microarray containing 125 cancer tissues. NSUN2 promoted nasopharyngeal carcinoma cell proliferation, migration, and invasion in vitro [107]. NSUN2 is upregulated in ATC and associated with dedifferentiation. Accordingly, knocking out NSUN2 in vivo and in vitro inhibited ATC formation, proliferation, invasion, and migration. Furthermore, inhibiting NSUN2 enhanced the sensitivity of ATC to genotoxic drugs [28]. Moreover, NSUN2 expression was significantly correlated with multi-drug resistance in ATC. NSUN2 regulated SRSF6 through ALYREF, induced alternative splicing reprogramming, and redirected the splicing form of the *UAP1* gene from AGX1 to AGX2. Therefore, AGX2 enhanced the N-linked glycation of ABC transporters, stabilizing them by preventing ubiquitination-mediated degradation. These results indicated that the NSUN2–SRSF6–UAP1 signaling axis is important in ATC multi-drug resistance [29].

NSUN2 is highly expressed in NSCLC tumor tissues, and the elevated expression level is closely related to tumor grading and size, accompanied by poor prognosis in patients with NSCLC. Knocking down NSUN2 in vitro and in vivo increased the sensitivity of NSCLC cells to ferroptosis activators. Furthermore, knocking down NSUN2 significantly inhibited *NRF2* mRNA and protein expression. Overall, NSUN2 maintained NRF2 expression through the m5C–YBX1 axis, promoting NSCLC progression [23]. Additionally, NSUN2 overexpression led to gefitinib resistance and tumor recurrence, while *NSUN2* gene inhibition caused tumor shrinkage in vitro and in vivo and overcame intrinsic resistance to gefitinib. That study revealed the key role of abnormal RNA m5C modification in mediating intrinsic resistance to gefitinib in *EGFR* mutant NSCLC through the NSUN2–YBX1–QSOX1 pathway [22]. NSUN2 and m5C methylation were highly expressed in LUAD samples, where NSUN2 facilitated LUAD malignancy progression by regulating m5C modifications to stabilize the PIK3R2 that activated PI3K–AKT signaling [24]. NSUN2 was significantly upregulated in hexavalent chromium-transformed cells and in the lung tissues of mice exposed to hexavalent chromium. Inhibiting NSUN2 reduced cell proliferation, migration, colony formation, and luminal formation abilities. NSUN2-mediated m5C modification triggered metabolic reprogramming and cell cycle changes. Additionally, knocking down NSUN2 weakened tumorigenesis and angiogenesis in the body. ALYREF was involved in NSUN2-mediated m5C modification in the hexavalent chromium-induced carcinogenic process [25].

NSUN2 is highly expressed in osteosarcoma tissues and cells, and high NSUN2 expression indicates poor prognosis in patients with osteosarcoma. NSUN2 upregulated FABP5 expression by increasing *FABP5* mRNA stability, promoting fatty acid metabolism in osteosarcoma cells and facilitating osteosarcoma progression [30].

In liver and gallbladder tumors, NSUN2 promotes tumor progression through multiple pathways. NSUN2 expression was significantly upregulated in HCC, and patients with HCC with high NSUN2 expression had a poorer prognosis. NSUN2 promoted HCC cell proliferation, migration, and invasion by regulating the Wnt signaling pathway. Knocking down NSUN2 significantly affected the abundance, distribution, and composition of m5C RNA modification in HCC cells [31]. HCC tissue samples contained upregulated NSUN2, SOAT2, and m5C levels. In vitro experiments revealed that NSUN2 enhanced energy metabolism reprogramming, inhibited CD8+ T cell activity and cytotoxicity, and promoted immune escape. Furthermore, in vivo studies confirmed the role of NSUN2 in promoting HCC immune escape and tumor formation by regulating the m5C modification of SOAT2 [32]. HCC tissues contained increased transcription levels of NSUN2 and FZR1. NSUN2 deficiency may inhibit cellular processes and tumor growth in HCC by suppressing FZR1 [108]. NSUN2 is also highly expressed in gallbladder carcinoma tissues and cell lines. NSUN2 promoted gallbladder carcinoma cell growth and colony formation by upregulating RPL6 [109].

In gastrointestinal malignancies, NSUN2 typically promotes tumor progression through multiple pathways. NSUN2 promotes GC cell proliferation, migration, and invasion in vitro, where it is upregulated in GC and predicts a poor prognosis. SUMO-2/3 stabilized NSUN2 and mediated its nuclear transport to directly interact with NSUN2, promoting the carcinogenic activity of NSUN2 [34]. NSUN2 is highly expressed in CRC and associated with poor survival in patients with CRC. NSUN2 promotes the growth of CRC cells in vivo and in vitro by stabilizing *SKIL* mRNA [35]. Downregulating NSUN2 limited CRC cell growth and induced ferroptosis, mainly through m5C methylation modification of SLC7A11 [36]. Furthermore, NSUN2 expression is significantly elevated and plays a carcinogenic role in CRC. The NSUN2–YBX1–m5C–ENO1 signaling axis leads to glucose metabolism reprogramming and increases in lactate, promoting CRC. Subsequently, lactic acid accumulation from CRC cells activates NSUN2 transcription through histone H3K18 lactylation and induces NSUN2 lactylation at Lys356 residue (K356), which is crucial for capturing target RNA [37]. The specific methylation of the tRNA Arg C34 site mediated by NSUN2 protected tRNA from endonuclease cleavage and promoted colon cancer cell metastasis in vivo and in vitro [110].

NSUN2 expression is elevated in prostate cancer tissues and cells, where NSUN2 contributes to prostate cancer cell proliferation and migration [111]. R-loops participate in regulating genomic stability and modulating gene expression, and are markedly elevated in bladder cancer cells relative to normal urothelial cells. NSUN2 promoted bladder cancer progression by facilitating PRDM11 epigenetic silencing by recruiting EZH2. Knocking out NSUN2 rendered tumors more sensitive to cisplatin, reducing tumor growth and increasing DNA damage levels, which was associated with reduced MRE11 recruitment to damage sites, impairing homologous recombination repair [40].

NSUN2 is significantly upregulated in endometrial cancer due to epigenetic enhancement of H3K4me3 levels in the promoter region, which is triggered by KDM5A downregulation. In vitro and in vivo, inhibiting the NSUN2–SLC7A11 axis suppressed tumor growth by increasing endometrial cancer cell lipid peroxidation and ferroptosis [41]. *NSUN2* RNA is upregulated in ovarian cancer and cervical cancer, with no significant difference observed in uterine corpus endometrial cancer. NSUN2 has no significant effect on cervical cancer proliferation, while it stabilizes *KRT13* mRNA through YBX1 to promote cervical cancer invasion and migration [48].

NSUN2 is upregulated in both UVM cells and tissues. Silencing NSUN2 inhibited UVM cell migration and suppressed cell proliferation through G1 arrest. Overexpressing miR-124a in UVM cells reduced the NSUN2 expression level, indicating that miR-124a is an upstream regulatory factor of this response. NSUN2-mediated CTNNB1 methylation status regulated UVM cell proliferation and migration [45]. Vitamin D3 significantly and specifically inhibited melanoma B16 cell proliferation and migration, enhanced vitamin D receptor expression, and reduced NSUN2 expression in melanoma cells. Overall, vitamin D3 inhibited the malignant progression of melanoma cells by binding to vitamin D responsive elements located upstream of the *NSUN2* promoter, reducing the transcriptional activity of NSUN2 [112].

NSUN2 expression is upregulated in DLBCL tissues and cells. Extracellular vesicles derived from DLBCL cells promoted the transfer of NSUN2 to DLBCL cells, promoting tumor cell proliferation, M2 macrophage polarization, and immune escape, while inhibiting apoptosis. Furthermore, the extracellular vesicle NSUN2 derived from DLBCL cells promoted tumor growth by positively regulating PDL1, stabilizing it in a YBX1-dependent manner, promoting tumor immune escape and M2 macrophage polarization [46]. NSUN2 was upregulated in AML and patients with high NSUN2 expression had a poor prognosis. Knocking down NSUN2 in AML cells inhibited cell proliferation and colony formation, and promoted apoptosis. Silencing NSUN2 in AML mice reduced the tumor burden and prolonged survival [42]. Functional studies have demonstrated that NSUN2 promotes leukemia cell proliferation, enhances tumor growth in xenograft models, and confers cell resistance to ferroptosis. The NSUN2–YBX1–FSP1 axis protects AML cells from ferroptosis stress by inhibiting lipid peroxidation and oxidative damage [43].

NSUN2 is upregulated in retinoblastoma and associated with poorer survival rates. Silencing NSUN2 prevented the malignant behavior of retinoblastoma cells. Downregulating NSUN2 inhibited glycolysis in retinoblastoma cells. Mechanistically, NSUN2 and YBX1 promoted the malignant behavior and in vitro glycolysis of retinoblastoma through HKDC1, and accelerated tumor growth in vivo [20]. NSUN2-mediated m5C RNA methylation promoted purine biosynthesis in the oncogenic process of retinoblastoma. Furthermore, NSUN2 expression was elevated in retinoblastoma samples and cell lines. In summary, the carcinogenic cascade reaction of NSUN2–ALYREF–m5C–PFAS is an important triggering factor for retinoblastoma [21].

Glucose is a cofactor that binds to NSUN2 amino acid positions 1–28, promoting its aggregation and activation. NSUN2 stabilizes TREX2 to limit cytoplasmic double-stranded DNA (dsDNA) accumulation and cGAS–STING activation, promoting tumorigenesis and resistance to PD-L1 immunotherapy. The above processes are closely related to apoptosis and CD8+ T cell infiltration, and reflect NSUN2 as a direct glucose sensor [51,113]. Lactate-mediated lactylation at the lysine 508 site enhances NSUN2 activity, which promotes GCLC. Activated GCLC induces higher levels of intracellular GSH, accompanied by reduced lipid peroxidation and the resistance phenotype to doxorubicin-induced GC cell ferroptosis. The NAA10–NSUN2–GCLC signal axis effectively counteracted ferroptosis under acidic conditions [52].

### 5.3. NSUN3 in Cancer

Upregulated NSUN3 expression in head and neck squamous cell (HNSC) indicated poor prognosis. Knocking down NSUN3 in vitro and in vivo inhibited HNSC proliferation and growth, increased M1 macrophage infiltration, and reduced the proportion of M2 macrophages [114].

NSCLC tissues contained elevated *NSUN3* mRNA levels. Functional studies have demonstrated that knocking down NSUN3 significantly inhibited A549 and PC9 cell viability and proliferation. Furthermore, NSUN3 deficiency enhanced CD8+ T cell cytotoxicity towards NSCLC cells and inhibited tumor growth in vivo. NSUN3 is a tumor driver in NSCLC pathogenesis that functions stably through *PDL1* mRNA [56].

NSUN3 is significantly overexpressed in HCC and associated with poor prognosis. NSUN3 promotes HCC cell proliferation by promoting ROS accumulation and activating the PI3K–AKT signaling pathway [11]. NSUN3 promoted CRC cell proliferation and migration. NSUN3 was specifically inhibited by Compound B19, a novel bipyridine derivative designed and synthesized based on the structure of caerulomycin A, which suppressed CRC progression [115].

The survival of patients with myodysplastic syndromes (MDS) with low NSUN3 expression was only 11 months, while the median survival of patients with high expression was close to 25 months [116].

### 5.4. NSUN4 in Cancer

NSUN4 is highly expressed in lung cancer tissues, consistent with the trend of circERI3 expression in lung cancer. The NSUN4 expression level correlated positively with the diameter of lung cancer, and the NSUN4-high expression group had a shortened OS. m5C microarray analysis determined that circERI3 contains the m5C modification, and NSUN4-mediated m5C modification of circERI3 increased its nuclear output, promoting lung cancer progression [61]. NSCLC tissues and cell lines contain upregulated NSUN4 and CDC20 levels. Inhibiting NSUN4 reduced NSCLC cell growth, stemness, migration, and invasion ability, while increasing NSUN4 had the opposite effect. CDC20 and NSUN4 expression were positively associated in NSCLC. Overall, NSUN4 promoted the activity of the oncogenic protein CDC20 by mediating m5C modification of *CDC20* mRNA, promoting NSCLC development [59].

NSUN4 is elevated in HCC tissues and cells, and activates the mTOR signaling pathway to promote HCC tumor progression in mice [117]. NSUN4 expression was significantly upregulated in HCC tissues and cell lines (Hep3B, Huh-7, HepG2, SMCC7721, MHCC97L) [118].

NSUN4 is highly expressed in glioma tissues and cells, and high m5C levels are negatively correlated with the prognosis of patients with glioma. Furthermore, NSUN4 enhances m5C modification of mRNA, promoting glioma malignant progression. NSUN4-mediated high m5C levels promoted ALYREF binding to *CDC42* mRNA and regulated its stability, thereby promoting glioma malignant progression [60].

### 5.5. NSUN5 in Cancer

Glioma cells feature *NSUN5* gene silencing associated with the high methylation of cancer-specific promoter CpG islands. NSUN5 has specific anti-cancer effects in gliomas. The absence of NSUN5 led to an unmethylated state at the C3782 site of 28S rRNA, which drives a decrease in overall protein synthesis and results in adaptive translation programs to maintain survival under cellular stress conditions. Most importantly, epigenetic inactivation of NSUN5 is a hallmark of long-term survival for patients with glioma [119]. NSUN5 downregulates β-catenin by promoting the degradation of its mRNA, enhancing the phagocytic activity of tumor-associated macrophages (TAMs) in gliomas. NSUN5–TET2–RBFOX2 signaling regulates RNA metabolism in gliomas [63]. Elevated NSUN5 expression is closely related to poor survival in patients with glioblastoma. Knocking down NSUN5 reduced protein synthesis, cell proliferation, spheroid formation, migration, and resistance to temozolomide in glioblastoma cell lines [64].

NSUN5 is significantly upregulated in esophageal cancer (ESCA) and has demonstrated good diagnostic potential. Enhancing NSUN5 expression accelerated ESCA cell proliferation, while knocking down NSUN5 weakened the ESCA cell proliferation ability and blocked the cell cycle in the G0/G1 phase. NSUN5 promoted ESCA through m5C modification of METTL1. Additionally, knocking down NSUN5 significantly inhibited gastric and colon cancer cell growth [65].

NSUN5 expression is upregulated in HCC tissues. NSUN5 promotes the HCC proliferation, invasion, and migration ability, indicating that *NSUN5* may be an important oncogene in HCC. Furthermore, high NSUN5 level enhanced the EMT process in HCC cells. Mechanistically, high-level NSUN5 promoted H3K4me3 enrichment in the *SMAD3* promoter region by recruiting WDR5, promoting HCC metastasis through the SMAD3-mediated EMT pathway [120]. NSUN5 expression is upregulated in HCC tissues and associated with adverse clinical outcomes. An elucidation of the carcinogenic role of NSUN5 in HCC development identified the ZBED3–Wnt–β-catenin signaling pathway as its downstream target [66]. EFNA3 and NSUN5 were both upregulated in HCC and associated with poorer survival. Silencing NSUN5 impeded HCC progression by inhibiting EFNA3-mediated m5C modified glycolysis [67]. NSUN5 expression is upregulated in choriocarcinoma tissue. NSUN5 overexpression enhanced the growth and metastasis of cholangiocarcinoma cells by positive regulation of glutaminase expression [68].

NSUN5 inhibits ferroptosis, and regulates the lipid ROS and Fe^2+^ levels in GC cells. NSUN5 and FTH1 have been positively correlated, and NSUN5 regulates FTH1 levels. Silencing NSUN5 or FTH1 inhibited the growth of SGC7901 tumors. The NSUN5–FTH1 axis promoted GC cell growth slightly by regulating erastin-dependent ferroptosis [121]. NSUN5 is overexpressed in GC tissue, positively correlated with tumor staging, and negatively correlated with patient prognosis. NSUN5 promoted GC cell in vitro proliferation, stemness, and migration, and in vivo growth, mainly by activating the WNT–β-catenin signaling pathway. Additionally, NSUN5 reduced CD8+ T cell infiltration in GC, promoting immune escape [122]. NSUN5 is also highly expressed in CRC, where its high expression indicates poor disease-free survival (DFS) in patients with CRC. NSUN5 promotes CRC cell proliferation and migration, and can render CRC cells resistant to doxorubicin in vivo and in vitro. NSUN5 upregulates BRCA2 and BRIP1 expression, and interacts with these proteins to prevent cell death under DNA damage [123]. Finally, NSUN5 upregulation in CRC cells was associated with advanced tumor stages (III, IV) and induced cell cycle arrest in vitro [124].

NSUN5 is upregulated in ccRCC tissues and renal cancer cell lines and is associated with poorer OS and progression-free survival (PFS). Silencing NSUN5 enhanced tumor cell aging while inhibiting cell proliferation and migration [125]. ENO3 is upregulated in ccRCC, while NSUN5 is involved in the ENO3-regulated Warburg effect and ccRCC cell progression. The NSUN5–ENO3 axis promoted ccRCC growth in vivo and in vitro [70]. NSUN5 is significantly overexpressed in prostate cancer tissues and positively correlated with advanced disease stages and poor prognosis. NSUN5 promoted prostate cancer proliferation and migration through the PI3K–AKT pathway, induced macrophage polarization into a pro-tumor phenotype, and promoted the formation of an inhibitory tumor microenvironment [126]. CDK13 expression is upregulated and fatty acid synthesis is increased in prostate cancer. The CDK13–NUN5–ACC1 pathway mediates fatty acid synthesis and lipid accumulation in prostate cancer cells, promoting prostate cancer progression [71].

### 5.6. NSUN6 in Cancer

Low NSUN6 expression is associated with poorer OS and DFS in ESCC. NSUN6-mediated tRNA m5C modification inhibits ESCC proliferation and migration by regulating E-cadherin [76].

NSUN6 expression is reduced in A549, PC9, H1299, and H1975 lung cancer cells. NSUN6 positively regulated NM23-H1 expression through m5C modification and inhibited lung cancer cell proliferation, migration, and EMT [77]. Furthermore, NSUN6 promoted breast cancer cell migration dependently on mRNA m5C modification [12,127].

HCC tissues and cell lines have significantly reduced NSUN6 expression levels. NSUN6 overexpression significantly inhibited the proliferation and migration ability of HCC cells in a PDX mouse model. Furthermore, knocking down the downstream NSUN6 target BMPER reversed the inhibitory effect of NSUN6 on HCC progression [78]. NSUN6 expression was downregulated in pancreatic cancer tissue samples, where NSUN6 expression correlated with clinical pathological parameters, including T staging and the Ki-67+ cell ratio. In vitro (a pancreatic cancer cell line) and in vivo (xenograft mouse model) studies have demonstrated that NSUN6 inhibited cell proliferation through the cell cycle. Furthermore, NSUN6 was effective for evaluating the tumor recurrence and survival of patients with pancreatic cancer [128].

In clinical practice, high NSUN6 expression is significantly associated with shorter DFS and OS in patients with colon cancer. NSUN6 is upregulated in both colon cancer tissues and cells. Mechanistically, NSUN6 promotes cell cycle progression and cell proliferation of colon cancer through the oncogene *METTL3* [79]. Immunohistochemistry has demonstrated increased NSUN6 expression in colon cancer, where high NSUN6 levels were associated with shorter OS. Furthermore, NSUN6 has been significantly associated with nerve invasion [129].

The loss of NSUN6 conferred resistance to susceptible glioma cell lines. NSUN6 affects both large and small RNA to control glioma response to therapy. NSUN6 m5C is lost in PDX with temozolomide-resistant glioblastoma. NSUN6 regulates mRNA stability in a time-dependent manner through m5C deposition and regulates the temozolomide response through NELFB-coordinated transcriptional pause. High NSUN6 expression yields survival benefits in glioblastoma and other cancers [130]. *NSUN6* is a key protective gene whose overexpression inhibits the proliferation and migration of LN229 and U251 glioma cell lines in vitro [131]. NSUN6 is upregulated in osteosarcoma, and higher NSUN6 expression indicates poor prognosis. Furthermore, NSUN6 led to osteosarcoma cell proliferation, migration, and invasion. NSUN6 promoted osteosarcoma progression through EEF1A2 expression and inhibiting the AKT–mTOR signaling pathway via m5C methylation [82]. MARCH8 ubiquitinated NSUN6 at the Lys271 and Lys462 sites, leading to proteasome degradation of NSUN6. The elevated ROS levels intensified NSUN6 ubiquitination and degradation by enhancing the interaction between NSUN6 and MARCH8. The NSUN6–m5C–YBX1–PEXs signaling axis regulates peroxisome biogenesis, ROS accumulation, and cisplatin sensitivity in osteosarcoma [83].

The abundance of m5C modification was higher in radiation-resistant cervical cancer samples, and the low NSUN6 expression in cervical cancer indicated sensitivity to radiotherapy and better prognosis. Conversely, NSUN6 overexpression has been associated with radiation resistance and poor prognosis in cervical cancer. Abnormal m5C high methylation and NSUN6 overexpression drive the radiotherapy resistance of cervical cancer, where elevated NSUN6 expression promotes radiation resistance by activating the NSUN6–ALYREF–m5C–NDRG1 pathway [84].

### 5.7. NSUN7 in Cancer

An immunohistochemical analysis determined that NSUN7-negative Ewing sarcoma was more resistant to treatment. NSUN7 immune activity and OS were significantly associated, where NSUN7-negative cases had a shorter OS than NSUN7-positive cases [132]. NSUN7 has an inhibitory effect on ccRCC proliferation, where decreasing NSUN7 levels increased the expression of CDK2 and CCNE1, and a decrease in S-phase cells was accompanied by an increase in the number of G2/M-phase cells [133]. The in vivo and in vitro experimental results confirmed that silencing NSUN7 significantly inhibited cervical cancer cell growth, spread, and metastasis while promoting apoptosis. Lastly, silencing NSUN7 significantly downregulated the key ErbB pathway proteins (HER2, STAT5, PI3K/phosphorylated PI3K) [134].

**Figure 2:**
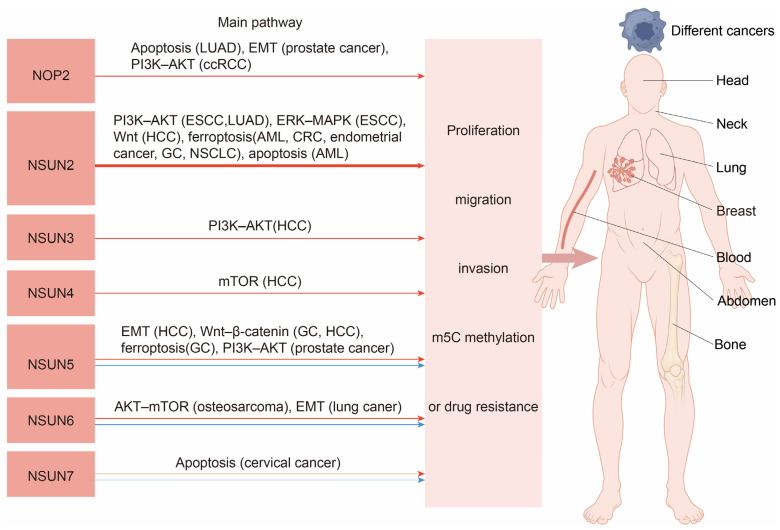
Schematic diagram of the main pathways through which the NSUN family genes influence cancer. The red arrow represents an increase, and the blue arrow represents a decrease.

The NSUN family genes exhibit diverse roles in cancer. Existing basic experiments indicate that NOP1, NSUN2, NSUN3, and NSUN4 predominantly promote the majority of cancers, while NSUN5, NSUN6, and NSUN7 demonstrate context-dependent effects—promoting some cancers but inhibiting others. Notably, NSUN7 has the least foundational research on cancer progression with only two studies, whereas NSUN2 has been the most extensively studied. Our analysis suggests that NSUN2 may function as a pan-cancer promoter in humans. Figure 2 illustrates the primary pathways through which NSUN family genes influence cancer. Table 2 summarizes the influence of the NSUN genes on cancer.

### 5.8. Pan-Cancer Differential Analysis of NSUN Family Genes

**Figure 3:**
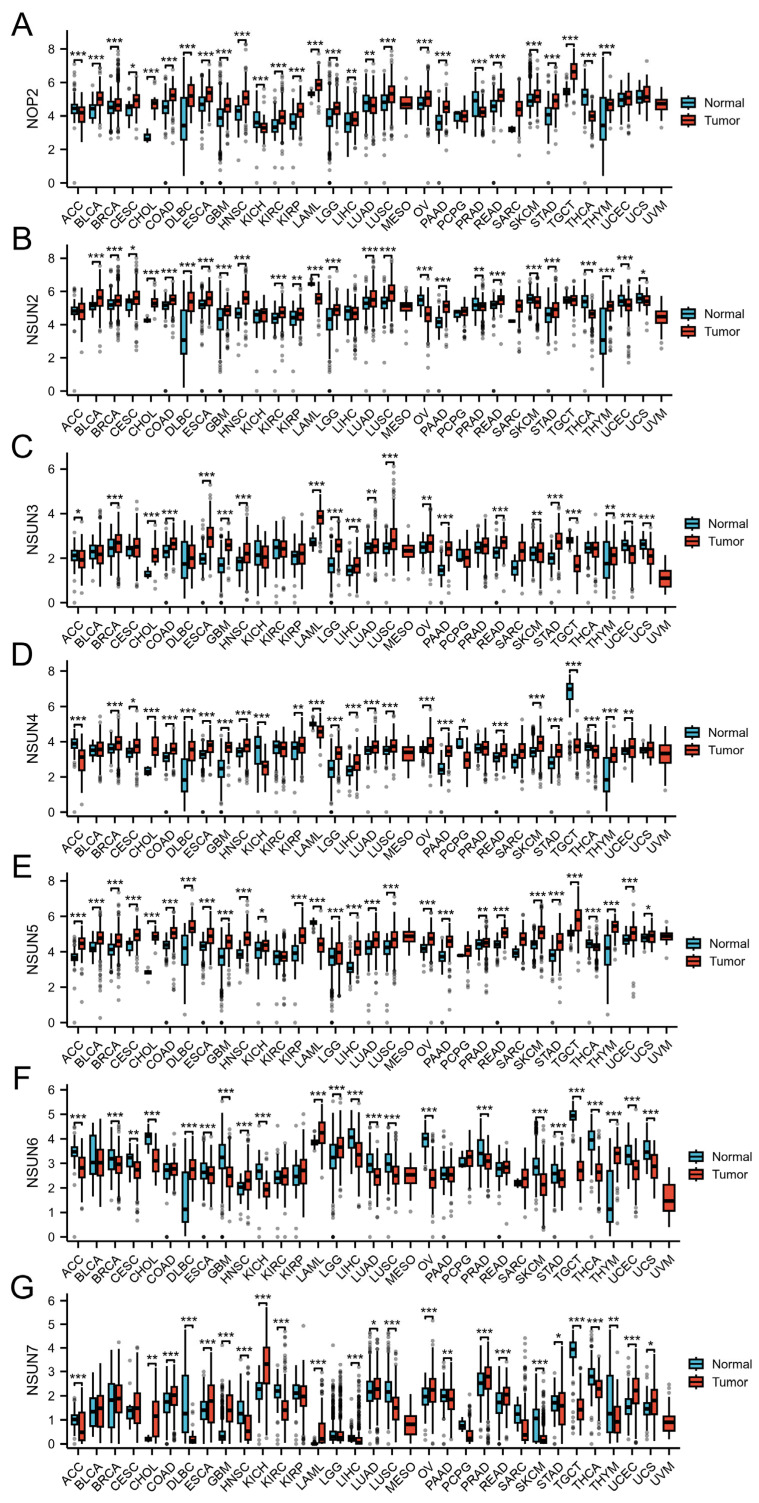
(**A**) NOP2, (**B**) NSUN2, (**C**) NSUN3, (**D**) NSUN4, (**E**) NSUN5, (**F**) NSUN6, and (**G**) NSUN7 expression in pan-cancer. * *p* < 0.05; ** *p* < 0.01; and *** *p* < 0.001; cholangiocarcinoma (CHOL); glioblastoma multiforme (GBM); lung squamous cell carcinoma (LUSC); ovarian serous cystadenocarcinoma (OV); skin cutaneous melanoma (SKCM); stomach adenocarcinoma (STAD); thymoma (THYM).

We grouped and compared the differences of NSUN family genes in public databases. The Cancer Genome Atlas (TCGA) database contains 33 tumors [135]. We directly analyzed the differences in molecules between multiple groups based on the Xiantao Online Database [(https://www.xiantaozi.com/, data source: TCGA + GTEx, TPM, processing: log2 (value + 1), URL (accessed on 30 August 2025)]. The statistical method used was the Mann–Whitney U test (Wilcoxon rank sum test). If there were cases where the sample size was <3 or the standard deviation (SD) within the data group was 0, these groups were not included in the statistical analysis (but would still be visualized). NOP2 demonstrated no differences in mesothelioma (MESO), pheochromocytoma and paraganglioma (PCPG), sarcoma (SARC), uterine corpus endometrial carcinoma (UCEC), uterine carcinosarcoma (UCS), and UVM among the 33 tumor types or <3 cases in normal tissues. NSUN2 demonstrated no differences in adrenocortical carcinoma (ACC), kidney chromophobe (KICH), liver hepatocellular carcinoma (LIHC), MESO, PCPG, SARC, testicular germ cell tumors (TGCT), and UVM, or <3 cases in normal tissues. NSUN3 demonstrated no differences in bladder urothelial carcinoma (BLCA), cervical squamous cell carcinoma, and endocervical adenocarcinoma (CESC), DLBCL, KICH, KIRC, KIRP, MESO, PCPG, prostate adenocarcinoma (PRAD), SARC, thyroid carcinoma (THCA), and UVM, or <3 cases in normal tissues. NSUN4 demonstrated no differences in BLCA, KIRC, MESO, PRAD, SARC, UCS, and UVM, or <3 cases in normal tissues. NSUN5 demonstrated no differences in KIRC, MESO, PCPG, SARC, and UVM, or <3 cases in normal tissues. NSUN6 demonstrated no differences in BLCA, colon adenocarcinoma (COAD), KIRC, KIRP, MESO, pancreatic adenocarcinoma (PAAD), PCPG, rectum adenocarcinoma (READ), SARC, and UVM, or <3 cases in normal tissues. NSUN7 demonstrated no differences in BLCA, breast invasive carcinoma (BRCA), CESC, KIRP, LGG, MESO, PCPG, SARC, and UVM, or <3 cases in normal tissues. The NSUN family genes demonstrated significant differences in most cancers. Figure 3 and Table 3 summarize the influence of the NSUN family genes on different cancers.

### 5.9. Pan-Cancer OS Analysis of NSUN Family Genes

**Figure 4:**
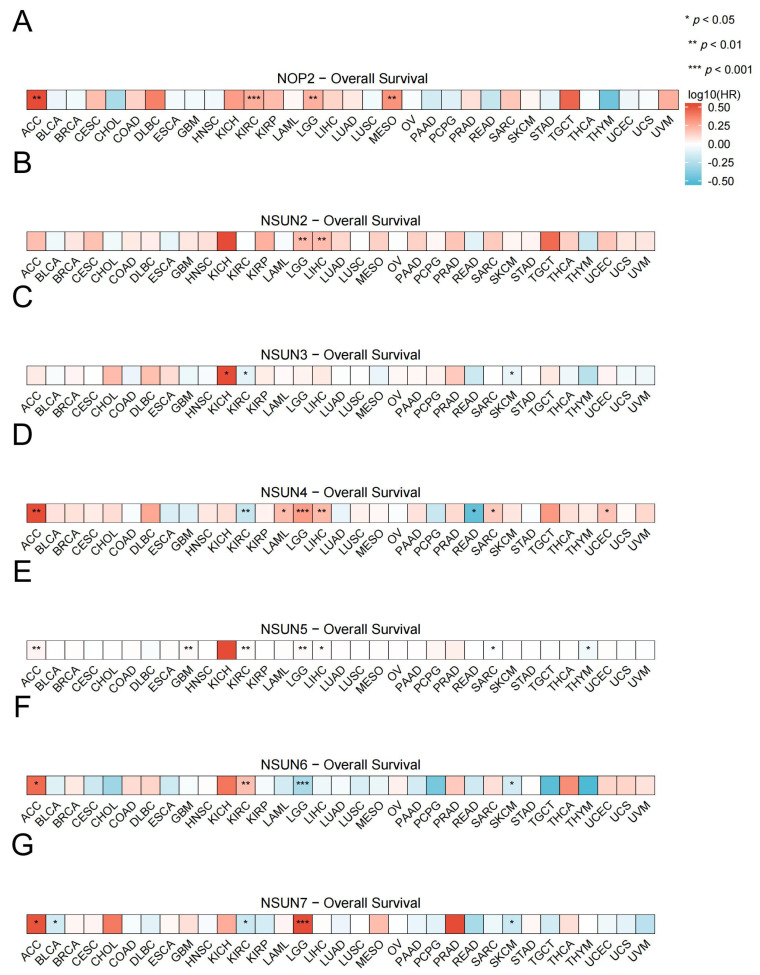
Pan-cancer overall survival analysis. (**A**) NOP2, (**B**) NSUN2, (**C**) NSUN3, (**D**) NSUN4, (**E**) NSUN5, (**F**) NSUN6, and (**G**) NSUN7 survival curves of pan-cancers between high and low expression groups. * *p* < 0.05; ** *p* < 0.01; and *** *p* < 0.001.

We created an OS prognostic heatmap for the NSUN family genes. Based on public data (TCGA, Xiantao Online Database), we directly analyzed the proportional regression risk model of molecules in diseases for prognostic data analysis, and visualized it with heatmaps [136]. The conditional assumption was that the observed values were independent, and the risk ratio did not change over time (proportional risk assumption). The patients with high NOP2 expression had a worse prognosis for ACC, KIRC, LGG, and MESO (Figure 4A). The patients with high NSUN2 expression had a worse prognosis for LGG and LIHC (Figure 4B). On the contrary, the patients with high NSUN3 expression had a worse prognosis for KICH, KIRC, and SKCM (Figure 4C). The patients with high NSUN4 expression had a worse diagnosis of ACC, LAML, LGG, LIHC, and UCEC, while the opposite was true for KIRC and READ (Figure 4D). The patients with high NSUN5 expression had a worse diagnosis of ACC, GBM, KIRC, LGG, and LIHC, while the opposite was true for SARC and THYM (Figure 4E). The patients with high NSUN6 expression had a worse diagnosis for ACC and KIRC, while the opposite was true for LGG and SKCM (Figure 4F). The patients with high NSUN7 expression had a worse prognosis for ACC and LGG, while the opposite was true for BLCA, KIRC, and SKCM (Figure 4G).

### 5.10. Pan-Cancer Immune Infiltration Analysis of NSUN Family Genes

Immune infiltration analysis: utilizing transcriptomic or other omics data, this method employs computational algorithms to estimate the fraction of immune cells in a tissue, thereby inferring the composition of immune cell populations within the tissue. We also analyzed immune infiltration using correlation heatmaps (TCGA, Xiantao Online Database), and analyzed the correlation between single genes and immune infiltration using public gene data [137,138]. Spearman analysis was used (non-parametric correlation tests do not require the data to meet the assumption of normality, offering broader applicability), and the results are presented as heatmaps. The analysis of the correlation between NSUN family genes and multiple immune infiltrates (Figures S1–S7) demonstrated that the NSUN family genes were correlated with various immune infiltrates (*p* < 0.05).

## 6. Role in Non-Tumorous and Non-Inflammatory Diseases

### 6.1. NOP2 (NSUN1) in Non-Tumorous and Non-Inflammatory Diseases

*Nop2* was an essential gene for development to the mouse blastocyst stage. Knocking down *Nop2* led to a global reduction in all RNA, including rRNA, small nuclear RNA, small nucleolar RNA, and mRNA. The results indicated that *Nop2* is an essential gene for RNA processing and/or stability during blastocyst formation in mouse preimplantation embryo development [6].

The lethality in homozygous animals indicates the important role of *Nop2*, but heterozygous animals enable the detection NOP2 expression in various tissues, including mouse brain. Histochemical, immunohistochemical, and immunoelectron microscopy techniques applied to mature mouse brains, human brains, and mouse neural stem cells have demonstrated that NOP2 is expressed in differentiated cells, including astrocytes, and in mature neurons. NOP2 has been detected in multiple regions of mouse and human brains, and is mainly concentrated in large pyramidal neurons. Two weeks after stroke, the number of NOP2/Nestin double-positive cells in the ischemic affected area and periventricular area were significantly increased. The newly discovered role of NOP2 in mature neurons and cells potentially involves neural tissue regeneration [139].

NOP2 binds to the T-cell factor (TCF)-binding element of the cyclin D1 promoter and activates its transcription. Removing NOP2 inhibited cyclin D1 promoter activity, leading to growth arrest and cell cycle distribution changes. NOP2 affected telomerase activation of cyclin D1 gene transcription, maintaining cell proliferation ability [140].

### 6.2. NSUN2 in Non-Tumorous and Non-Inflammatory Diseases

NSUN2 inactivation-knockout mice, patient-derived fibroblasts, and CRISPR/Cas9 knockout in human cell models have indicated that NSUN2 is necessary for generating m5C at positions 48, 49, and 50 of several mammalian mitochondrial tRNAs [141].

Whole-exome sequencing has demonstrated a homologous mutation (c.1020delA) in the *NSUN2* gene, which is caused by a frameshift and premature stop codon and leads to decreased *NSUN2* mRNA levels in children and may also cause intellectual disability [142]. The G679R NSUN2 mutant located in the nuclear cytoplasm is associated with intellectual disability and reduces NSUN2-mediated tRNA m5C in human cell lines and fruit flies. The tRNA m5C level is positively correlated with the cognitive performance of fruit flies, with G679R-NSUN2 expression leading to the most severe social behavior deficits. An NSUN2 variant lacking an internal intrinsically disordered region expression led to milder deficits [143]. NSUN2 expression and RNA m5C levels were significantly increased during the cerebral epithelial wound healing process. Knocking out NSUN2 significantly delayed in vivo cerebral epithelial wound healing and inhibited human cortical epithelial cell proliferation and migration, while overexpressing NSUN2 significantly enhanced proliferation and migration. Lastly, NSUN2 mainly promoted UHRF1 translation by recruiting the RNA m5C reader ALYREF [44].

Whole-exome sequencing also revealed a previously unreported homozygous nonsense variant located in exon 9 of *NSUN2* (NM_017755.5: c.1004T>A, p.Leu335*). This NSUN2 variation is related to the following symptoms: feeding difficulties, slender hands and fingers, severely restricted finger mobility, hallux valgus, varus foot, and elevated α-hydroxybutyrate dehydrogenase [144].

NSUN2 expression was significantly elevated in the hypertrophic cardiomyocytes of humans, rats, and mice. Knocking out *Nsun2* eliminated the hypertrophic response of mice to various stresses and accelerated heart failure progression. NSUN2 promotes the translation of *PRKACA*. The absence of NSUN2 significantly weakened PKA signaling pathway activation, impaired myocardial cell contraction and relaxation, and transiently interfered with calcium. Furthermore, overexpressing NSUN2 and PRKACA 3′UTR transcripts in the myocardium, respectively, sensitized and desensitized the heart to the hypertrophy response [54].

*Nsun2* deficiency created a suitable mouse model for studying tsRNA, which improved cell proliferation and survival under stress. The observation of mild liver injury in the *Nsun2* knockout mice compared to WT mice in short- and long-term liver injury suggested that the *Nsun2* deficiency alleviated liver injury in vivo [145]. The NSUN2 protein level was significantly reduced in liver cell ferroptosis. This result was attributed to STUB1-mediated ubiquitination of NSUN2 at lysines 457 and 654, which promoted NSUN2 degradation in ferroptosis. The reduction of m5C methylation of *GPX4* mRNA mediated by NSUN2 disrupted the interaction between SBP2 and SECIS, inhibiting GPX4 protein expression. Lower GPX4 expression promoted liver cell ferroptosis in vivo and in vitro, while restoring NSUN2 reversed this situation. These results indicated that the STUB1–NSUN2–GPX4 axis regulates liver cell ferroptosis [33]. High blood sugar and abnormal lipid metabolism are typical features of type 2 diabetes mellitus. NSUN2 expression was significantly upregulated in clinical samples of type 2 diabetes mellitus and mice fed a high-fat diet. Knocking down NSUN2 enhanced glucose tolerance and pyruvate metabolism, improved insulin resistance, and inhibited liver lipid accumulation in the high-fat diet-fed mice. Overall, NSUN2 mediates the dysregulation of liver glucose and lipid metabolism during type 2 diabetes mellitus by relying on m5C-mediated ACSL6 [9].

Knocking down NSUN2 increased 3T3-L1 preadipocyte lipid accumulation by accelerating cell cycle progression during mitotic clonal expansion at the early stage of adipogenesis. As the *CDKN1A* translation decreased, it accelerated the cell cycle and promoted fat production [47].

NSUN2 is an active regulator of endothelial inflammation. The lack of donor *Nsun2* impaired graft arteriosclerosis formation in a rat aortic graft model [49]. T cell infiltration in the vascular wall decreases in the absence of *Nsun2*. *Nsun2* deficiency significantly alleviated elastase-induced and hyperhomocysteinemia-aggravated mouse abdominal aortic aneurysm. *Nsun2* mediated the development of hypothalamic aortic aneurysm exacerbated by hyperhomocysteinemia by increasing the expression, secretion, and T cell migration of endothelial ATX [146].

NSUN2 is highly expressed in embryonic stem cells and is crucial for multiple biological processes, including regulating the balance between pluripotency and differentiation, and brain development. The specific endogenous enhancer of *Nsun2* in embryonic stem cells has been identified and is involved in enhancer-mediated gene regulation in the cells. Knockdown of the enhancer RNA (eRNA) was associated with decreased *Nsun2* expression, reduced m5C methylation activity, and decreased expression of multipotent markers [147]. Hu et al. reported that the NSUN2–JARID2–ALYREF axis coordinates histone modifications and chromatin accessibility, supporting normal fetal development [55].

Glucose can bind to conserved sequences in NSUN2, enhancing its binding with S-adenosyl-L-methionine and increasing its enzymatic activity, promoting cell differentiation. Additionally, glucose enhances the function of NSUN2, including promoting the translation of key pro-differentiation mRNAs such as *GRHL3* containing the m5C modification [148].

### 6.3. NSUN3 in Non-Tumorous and Non-Inflammatory Diseases

*Nsun3* mutant cells demonstrated significantly reduced methylation and formylation of mitochondrial tRNAMet (mt-tRNAMet), and decreased mitochondrial translation and respiratory capacity. The results indicated that *Nsun3* regulates embryonic stem cell differentiation by promoting mitochondrial activity [149]. Mouse embryos with whole-body *Nsun3* knockout died between embryonic day E10.5 and E12.5, demonstrating the importance of *Nsun3* for mouse embryo development. Heart-specific *Nsun3* knockout (Nsun3^HKO^) mice were generated to determine the function of NSUN3 in adult tissues. The cardiac mitochondria of the Nsun3^HKO^ mice were enlarged and contained fragmented cristae. Nsun3^HKO^ enhanced cardiac contraction and age-related mild cardiac enlargement. Furthermore, the hearts of Nsun3^HKO^ mice did not demonstrate downregulated mitochondrial mRNA encoding respiratory complex subunits, but demonstrated decreased respiratory complex enzyme activity, especially in older mice. The results indicated that NSUN3-mediated mitochondrial tRNA 5-formylcytidine modification is crucial for mouse embryonic development and respiratory complexes [150].

Patients with *NSUN3* rs7653521 CT and TT/CT genotypes exhibit a significantly lower risk of neuroblastoma compared to patients with the rs7653521 CC genotype. Additionally, carriers with three protective genotypes have a much lower risk of developing neuroblastoma than patients with 0–2 protective genotypes [151].

Whole-exome sequencing identified a functional loss mutation of *NSUN3* in a patient exhibiting combined mitochondrial respiratory chain complex deficiency. The results indicated that NSUN3 is necessary for efficient mitochondrial translation and revealed that oxidative processing of m5C generates 5-formylcytosine in human mitochondrial RNA [152].

Nakano et al. demonstrated that 5-formylcytidine C34 biosynthesis is initiated by S-adenosylmethionine (AdoMet)-dependent methylation reaction catalyzed by NSUN3, a hypothesized mitochondrial methyltransferase. NSUN3 knockout cells exhibited significantly decreased mitochondrial protein synthesis and oxygen consumption, leading to insufficient mitochondrial activity. NSUN3-mediated m (5) cytochrome 34 (C34) formation initiated the biosynthesis of f (5) C34 [153].

A patient with early-onset mitochondrial encephalomyopathy and epilepsy possessed the novel biallelic *NSUN3* missense variants c.421G>C (p.A141P) and c.454T>A (p.C152S) [154]. NSUN3 is located in the mitochondria and interacts with mt-tRNAMet to methylate C34 at the rocking site. Both *NSUN3* and *ABH1* deletions severely affect mitochondrial translation in human cells, indicating that the modifications generated by these two enzymes are necessary for mt-tRNAMet function [155]. The pathogenic or potentially pathogenic biallelic variants in *NSUN3* disrupt mt-tRNAMet methylation and mitochondrial translation, leading to mitochondrial diseases that range from mild isolated optic atrophy to severe multi-system phenotypes, which may be accompanied by limited life expectancy [156]. Genetic testing determined that a child with bilateral optic nerve atrophy and nystagmus had a homozygous variant c.349_352dup p.(Ala118Glufs*45) in *NSUN3*, indicating familial segregation consistent with autosomal recessive inheritance. Additional functional analysis revealed a decrease in *NSUN3* mRNA levels in the patient’s fibroblasts, a slight decrease in mitochondrial complex IV levels, and a lower cellular respiration rate compared to the healthy control group [157].

NSUN3 (maintains mitochondrial function through m5C → f5C modification along with ALKBH1) is abnormally overexpressed in the reticulocytes of hemoglobin H Disease-Constant Spring patients. Functional experiments have demonstrated that NSUN3 overexpression significantly enhanced the accumulation of intracellular ROS and malondialdehyde, decreasing GSH levels and weakening the overall cellular antioxidant capacity (T-AOC). This result may have been due to the accumulation of ROS, which inhibit the synthesis of mitochondrial respiratory chain complexes I, II, and IV, leading to abnormal m5C → f5C modification. Additionally, overexpressing NSUN3 suppresses Nrf2 phosphorylation, exacerbates oxidative stress, hinders its transfer to the nucleus, and weakens the cellular antioxidant system. Furthermore, NSUN3 overexpression exacerbated intracellular DNA damage and inhibited cell proliferation activity, while silencing NSUN3 yielded the opposite result [158].

### 6.4. NSUN4 in Non-Tumorous and Non-Inflammatory Diseases

MTERF4 regulates translation by targeting the methyltransferase NSUN4 to mammalian mitochondrial ribosomes [159]. NSUN4 is a key regulatory factor of mitochondrial double-stranded RNA (mt-dsRNA) expression in human cells and induces m5C modification on mtRNA, especially at the end of light chain non-coding RNA. Inhibiting NSUN4 can increase mt-dsRNA expression [160]. mt-dsRNA can spontaneously form in the mitochondria, hindering mitochondrial gene expression and triggering immune responses. NSUN4-mediated RNA methylation (m5C) recruits RNA degradation mechanisms to prevent dsRNA formation [161]. The complex formed by NSUN4 and MTERF4 is essential in mitochondrial ribosome biosynthesis, as conditional *Nsun4* mouse gene knockout completely inhibited mitochondrial translation. Furthermore, NSUN4 is a bifunctional protein that is required for 12S rRNA methylation, and also interacts with MTERF4 to promote monoribosome assembly [162].

Silencing *Nsun4* reduced m5C levels in bone marrow-derived mesenchymal stem cells (BMSCs). These data indicated that m5C methylation is dependent on *Nsun4* during cartilage differentiation. The *Nsun4*- and *Mettl3*-mediated *Sox9* translation reprogramming promoted BMSC chondrogenic differentiation [62].

### 6.5. NSUN5 in Non-Tumorous and Non-Inflammatory Diseases

Niujiao Dihuang Jiedu decoction has a significant therapeutic effect on acute-on-chronic liver failure, where it inhibited acute-on-chronic liver failure-related ferroptosis by promoting SLC7A11 m5C methylation, which is generated by binding to NSUN5 [72]. The NSUN5–FTH1–FTL pathway mediates BMSC ferroptosis, and targeting the components of this pathway may promote resistance to ferroptosis and improve the survival rate of transplanted BMSCs [163]. Overexpressing *Nsun5* inhibited macrophage M1 polarization and osteoclast differentiation, protected bone mass in ovariectomized mice. CX3CL1 accelerated M1 macrophage polarization and promoted osteoclast differentiation, regulated by NSUN5-mediated m5C modification [73].

Single-gene Nsun5-KO mice exhibited reduced cortical development, leading to spatial cognitive deficits [164]. The corpus callosum volume of the Nsun5-KO mice decreased on day 60 after birth, the number of myelin axons decreased, and the myelin sheath was relaxed. The absence of *Nsun5* inhibited the proliferation of oligodendrocyte precursor cells [165].

*Nsun5*−/− mice exhibited weight loss compared to WT mice. Additionally, their survival rate gradually decreased to 20% on day 21 after birth. Further examination demonstrated that the *Nsun5*−/− mice had multi-organ damage, with the kidneys being the most severely damaged. Mice lacking NSUN5 died before puberty due to fatal kidney damage and apoptosis. These results indicated that NSUN5 is crucial in preventing the accumulation of DNA damage and apoptosis in the kidneys [166].

The lack of *Nsun5* hindered follicular development and ovarian function, directly inhibiting embryogenesis and embryonic development [75]. *NSUN5* mutations may alter decidualization through the L-11Rα–JAK2–STAT3–cyclin D3 pathway, affecting placental development and promoting the occurrence of preeclampsia [167]. The decidua tissue of women with severe gestational hypertension had significantly downregulated NSUN5 expression compared to the decidua tissue of normal pregnant women. NSUN5 expression also increased when decidualization was induced in vitro. ATP content decreased during the process of cell decidualization and after NSUN5 had been knocked down. NSUN5 may interact with ATP5B and affect preeclampsia [168]. *Nsun5* is upregulated at the two-cell stage of mouse preimplantation development. Knocking down *Nsun5* caused significant developmental disorders, including reduced blastocyst formation, decreased blastocyst volume, and obstructed hatching from the zona pellucida. Furthermore, *Nsun5* knockdown decreased the number of embryonic cells and increased apoptosis, decreased YAP1 nuclear translocation, and upregulated the Hippo pathway [169].

The decrease in NSUN5 levels increased the lifespan and stress resistance of yeast, nematodes, and fruit flies [170]. The NSUN5 N-terminal domain is essential for localization to the nucleolus, while two evolutionarily highly conserved cysteine residues mediate catalytic reactions. In mammalian cells, the phenotypic consequences of NSUN5 deficiency include reduced proliferation and cell size, which can be attributed to a decrease in total protein synthesis caused by ribosomal changes. In mice, *Nsun5* knockout decreased their body weight and lean body mass, while their food intake remained unchanged, and a trend of reduced protein synthesis was observed in several tissues [171]. Researchers have provided evidence of the effects of *Nsun5* on the structure of the primary somatosensory cortex and reported that a lack of *Nsun5* can disrupt pain-related behaviors [172].

Four potential tetralogy of Fallot mutations in the *NSUN5* coding region have been identified. The cardiac outflow tract did not develop normally in *Nsun5*-deficient mouse embryonic hearts. The misalignment of the aorta and diaphragm defects were due to delayed fusion of the membranous ventricular septum, which delayed the development of the cardiac outflow tract. The loss of NSUN5 function impairs the m5C modification and translation efficiency of important cardiac genes [173]. m5C expression is significantly higher in abdominal aortic aneurysm than in normal aortic samples. NSUN2 and NSUN5 were upregulated at both the protein and mRNA level in abdominal aortic aneurysm tissues [174].

### 6.6. NSUN6 in Non-Tumorous and Non-Inflammatory Diseases

NSUN6 expression was significantly decreased in the white matter and superior gyrus in Alzheimer disease individuals [175]. The biallelic pathogenic variant in *NSUN6* leads to an autosomal recessive intellectual disability [176].

NSUN6 is a methyltransferase that can methylate C72 of tRNAThr and tRNACys and acts as an mRNA methyltransferase targeting the Type II m5C site. Furthermore, an NSUN6 variant that has lost its tRNA methylation ability but retained its mRNA methylation ability has been identified [177]. NSUN6 mainly targets the 3′UTR of the consensus sequence motif CTCCA, which is located in the loop of the hairpin structure. Knockout and recovery experiments have demonstrated that mRNA and translation levels were enhanced when NSUN6-targeted mRNA was methylated. Ribosomal analysis further indicated that NSUN6-specific methylation is associated with translation termination [127]. Methylation is closely related to fat, metabolism, inflammation, and other factors [178]. Chronic intermittent hypoxia can increase NSUN6 levels in adipose tissue. NSUN6 is believed to be involved in macrophage ferroptosis and M1 polarization. NSUN6 inhibited M2 macrophage recruitment through m5C methylation [85].

Serum stable carotid atherosclerotic plaque patients had a higher *NSUN6* gene expression level than unstable patients, and HTR7 demonstrated the opposite trend. The pro-inflammatory factors TNF-α and IL-6 were reduced in macrophages overexpressing *NSUN6* or in which *HTR7* had been knocked down [179]. Furthermore, RT-qPCR and Western blot confirmed significant NSUN6 upregulation in a rat model of heart failure [180]. Under certain conditions, *Nsun6* deficiency may affect the immune response of the mouse spleen and the redox response of the liver, and is also associated with the formation of antibody-secreting plasma cells [181].

The absence of NSUN2 or NSUN6 reduced HEK293T cell proliferation and increased the expression of the aging-related marker P27, while more β-galactosidase-positive cells were observed in H_2_O_2_-induced oxidative stress response. Additionally, NSUN2 and NSUN6 expression in the HGPS premature aging cell line was significantly reduced due to LMNA^G609G^ mutation [182].

### 6.7. NSUN7 in Non-Tumorous and Non-Inflammatory Diseases

NSUN7 involved in regulating the longitudinal column positioning in sperm flagella. NSUN7 may have an essential function in the spermatogenesis of mice independent of its methyltransferase activity [183]. T26248G transversion mutation in exon 7 of the *Nsun7* gene led to changes in protein folding, which was associated with decreased sperm motility in men with asthenozoospermia. The abovementioned *Nsun7* mutation could be used as an inflammatory marker in asthenospermic men [184]. NSUN7 is a protein present in elongated sperm cells and interacts with RNA specific to the precursor cells of such sperm cells. *Nsun7* gene inactivation in mice led to the upregulation of its RNA interactors, indicating that *Nsun7* downregulates a group of RNAs in elongated spermatids. The physiological consequence of *Nsun7* gene deletion is male infertility, which is caused by the abnormal position of the observed longitudinal column relative to the axonal microtubule dyad, leading to motor defects [185]. The NSUN7 in Alzheimer disease individuals in the hippocampus was significantly increased [175].

## 7. Conclusions and Future Prospects

m5C is a nucleic acid modification widely present in various RNAs, including tRNA, rRNA, mRNA, eRNA, lncRNA, circRNA, and miRNA. m5C is a reversible epigenetic modification that is crucial in RNA metabolism, affecting RNA stability, nuclear output, and translation processes [113]. Decreased NSUN2 levels improved the translation efficiency of a large group of specific mRNAs, including shorter mRNAs with higher GC content, where motifs with C residues are located in GC-rich environments. The next research frontier in this field may be the discovery of the mechanisms by which NSUN2 affects cellular pathways related to mRNA packaging [8]. NSUN3 is closely related to mitochondrial diseases and cancer. Abnormal NSUN family genes are closely related to various diseases. The information provided in this article confirmed their contributions in the following areas: neurological diseases, viral and bacterial infections, and their influence on tumor progression. In clinical body fluid samples, they can serve as potential diagnostic genes; however, larger sample sizes and multi-center experiments are required to verify its reliability.

This NSUN family gene review is subject to limitations. Although significant progress has been made in NSUN family gene research, the following aspects should be explored further: the mechanism research is imperfect, the role of m5C modification in carcinogenesis is not fully understood, the *NSUN2* gene significantly influences cancer research most studies have indicated that *NSUN2* promotes the progression of various cancers, and there is relatively little research on other genes in the family. In particular, the precise mechanism of action of different NSUN members on specific RNAs requires further exploration. The limitations of the research model are as follows: some studies only used cell line experiments and did not fully reflect the heterogeneity of patient tumors. The clinical application challenges include the fact that the small molecule inhibitors targeting NSUN2 and other genes remain in the early stages of development [186], and specificity still requires optimization. Regarding cross-species differences, some studies are based on animal models, and the functions of NSUN family members may vary among different species, which increases the difficulty of translating research results. Regarding technical limitations, the sensitivity and accuracy of m5C-modified sequencing technology can be improved further.

If precise detection, synergistic mechanisms with other epigenetic modifications, and optimized targeted intervention strategies for the precise treatment of diseases while minimizing damage to normal cell physiology can be solved, NSUN family gene research can produce more new breakthroughs in disease diagnosis and treatment.

## Figures and Tables

**Table 1:** Methylation relationship among NSUN family genes, other genes and functions.

NSUN Family	Genes Related to Methylation	Functions Related to Methylation
NOP1	APOL1 [15]; c-Myc, EIF3A, LDHA, TPI1, PKM2, and ENO1 [13]; EZH2 [14]; LMNB2 [18]; RAPGEF4 [5]; TAR [16]; XPD [17]	Promotes glycolysis [13]; activates PI3K-Akt pathway in ccRCC [15]; suppresses HIV-1 transcription [16]
NSUN2	ACSL6 [9]; c-Myc, BCL2, RAB31, JUNB, and TRAF2 [28]; CDKN1A [47]; CTNNB1 [45]; FABP5 [30]; FSP1 [43]; GCLC [52]; GPX4 [33]; HKDC1 [20]; ICAM-1 [49]; Il17a and Il17f [38]; IL-17A [50]; IRF3 [53]; Jarid2/Ezh2 [55]; KRT13 [48]; LIN28B and GRB2 [27]; ME1, GLUT3, and CDK2 [25]; NRF2 [23]; PIK3R1 and PCYT1A [34]; PDL1 [46]; PFAS [21]; PHGDH and SHMT2 [42]; PIK3R2 [24]; PINK1 [10], PRKACA [54]; PTPRD [19]; QSOX1 [22]; SARS2 [31]; SKIL [35]; SLC7A11 [36,41]; SMOX [26]; SOAT2 [32]; SRSF6 [29]; TREX2 [51]; UHRF1 [44]	Exacerbates inflammation-mediated tissue damage after traumatic brain injury [19]; metabolic reprogramming and cell cycle changes in lung cancer [25]; transports amino acids, especially leucine [28]; promotes fatty acid metabolism in osteosarcoma [30]; reprograms glucose metabolism and lactate mechanisms in CRC [37]; R-loop stabilization of bladder cancer [40]; increases leukocyte adhesion to endothelial cells [49]; hyperhomocysteinemia [50]; glucose and lipid metabolism dysregulation in the liver [9]; regulation in the cardiac hypertrophic program [54]; precisely orchestrates fetal development [55]
NSUN3	PD-L1 [56]; TAK1 [58]; TIFA [57]	Reduces CD8+ T-cell-mediated cytotoxicity against NSCLC cells [56]; aggravates sepsis-associated acute kidney injury [57]; promotes sepsis-induced pulmonary injury [58]
NSUN4	CDC20 [59]; CDC42 [60]; circERI3 [61]; Sox9 [62]	Affects mitochondrial function and energy metabolism of lung cancer [61]; promotes the repair of cartilage defects [62]
NSUN5	ACC1 [71]; CTNNB1, TET2 and RBFOX2 [63]; CX3CL1 [73]; EFNA3 [67]; ENO3 [70]; GLS [68]; GPX4 [69]; METTL1 [65]; SLC7A11 [72]; ZBED3 [66]	Accumulation of intracellular glutaminase [68]; activates the cGAS-STING pathway in COAD [69]; promotes lipid deposition [71]; resistance against ferroptosis [72]
NSUN6	BMPER [78]; CDH1 [76]; EEF1A2 [82]; HDAC10 [85]; METTL3 [79]; NDRG1 [84]; NM23-H1 [77]; PEX1 and PEX3 [83]	Affects Akt–mTOR signaling pathway in osteosarcoma [82]; inhibits the recruitment of M2 macrophages [85]
NSUN7	CCDC9B [86]; NLRP3 [87]	Affects pyroptosis of granulosa cells [87]

**Table 2:** Influence of NSUN family genes on cancer.

Cancer	NOP2	NSUN2	NSUN3	NSUN4	NSUN5	NSUN6	NSUN7
Retinoblastoma		Promote proliferation [21] and migration [20]					
Nasopharyngeal carcinoma		Promote proliferation, migration and invasion [107]					
ATC		Promote proliferation, migration and invasion [28]					
ESCA		Promote proliferation, migration and invasion [26,27]			Promote proliferation [65]	inhibit proliferation and migration [76]	
HNSC			Promote proliferation [114]				
NSCLC		Promote gefitinib resistance [22]. Promote proliferation, migration and invasion [23]	Promote proliferation [56]	Promote proliferation, migration and invasion [59]			
LUAD		Promote proliferation, migration and invasion [24,25]					
Lung cancer	Promote migration and invasion [14,102]			Promote proliferation [61]		inhibit proliferation and migration [77]	
Breast cancer						Promote migration [12,127]	
HCC	Promote proliferation, migration and invasion [13],	Promote proliferation, migration and invasion [31,32,108]	Promote proliferation [11]	Promote proliferation, migration and invasion [117]	Promote proliferation [66,67], migration and invasion [120]	inhibit proliferation and migration [78]	
gallbladder carcinoma		Promote proliferation [109]					
cholangiocarcinoma					Promote proliferation, migration and invasion [68]		
pancreatic cancer						inhibit proliferation [128]	
GC	Promote proliferation [103]	Promote proliferation, migration and invasion [34]			Promote proliferation [65,121] and migration [122]		
CRC	Promote proliferation, migration and invasion [18,104]	Promote proliferation [36], migration [35] and invasion [37,110]	Promote proliferation and migration [115]		Promote proliferation [65,124], migration and Doxorubicin Resistance [123]	Promote proliferation [79]	
ccRCC	Promote proliferation, migration and invasion [15]				Promote proliferation [70] and migration [125]		inhibit proliferation [133]
prostate cancer	Promote invasion [106]	Promote proliferation and migration [111]			Promote proliferation [71] and migration [126]		
bladder cancer		Promote proliferation, migration and cisplatin resistance [40]					
high-grade serous ovarian cancer	Promote proliferation, migration and invasion [5]						
endometrial cancer		Promote proliferation [41]					
cervical cancer		Promote migration, invasion [48]					Promote proliferation, migration and invasion [134]
osteosarcoma		Promote proliferation, migration, invasion [30]				Promote proliferation, migration and invasion [82], cisplatin resistance [83]	
UVM		Promote proliferation and migration [45]					
melanoma		Promote proliferation and migration [112]					
glioma				Promote proliferation and migration [60]	inhibit proliferation [63,119]	inhibit proliferation and migration [131]	
glioblastoma					Promote proliferation, migration and temozolomide resistance [64]		
AML		Promote proliferation [42,43]					
DLBCL		Promote proliferation [46]					

**Table 3:** Up- or down-regulating NSUN family genes in pan-cancer (*p* < 0.05).

Cancer Type	NOP2	NSUN2	NSUN3	NSUN4	NSUN5	NSUN6	NSUN7
ACC	down		down	down	up	down	down
BLCA	up	up			up		
BRCA	up	up	up	up	up	down	
CESC	up	up		up	up	down	
CHOL	up	up	up	up	up	down	up
COAD	up	up	up	up	up		up
DLBC	up	up		up	up	up	down
ESCA	up	up	up	up	up	down	up
GBM	up	up	up	up	up	down	up
HNSC	up	up	up	up	up	up	down
KICH	down			down	up	down	up
KIRC	up	up					down
KIRP	up	up		up	up		
LAML	up	down	up	down	down	up	up
LGG	up	up	up	up	up	up	
LIHC	up		up	up	up	down	down
LUAD	down	up	up	up	up	down	up
LUSC	up	up	up	up	up	down	down
OV	up	down	up	up	up	down	up
PAAD	up	up	up	up	up		down
PCPG				down			
PRAD	down	down			up	down	up
READ	up	up	up	up	up		up
SARC		up					
SKCM	up	down	up	up	up	down	down
STAD	up	up	up	up	up	down	down
TGCT	up		down	down	up	down	down
THCA	down	down		down	down	down	down
THYM	up	up	up	up	up	up	down
UCEC		down	down	up	up	down	up
UCS		down	down		up	down	up

## Data Availability

The data that support the results of current study is available on TCGA and other databases. The datasets used and/or analyzed during the current study are available from the corresponding author on reasonable request.

## Supplementary Materials

The following supporting information can be downloaded at: https://www.mdpi.com/article/10.3390/biomedicines13122951/s1, Figure S1: NOP2 expression correlation with immune infiltration; Figure S2: NSU·N2 expression correlation with immune infiltration; Figure S3: NSUN3 expression correlation with immune infiltration; Figure S4: NSUN4 expression correlation with immune infiltration; Figure S5: NSUN5 expression correlation with immune infiltration; Figure S6: NSUN6 expression correlation with immune infiltration; Figure S7: NSUN7 expression correlation with immune infiltration.

## Abbreviations

The following abbreviations are used in this manuscript:

ACC	Adrenocortical carcinoma
AML	Acute myeloid leukemia
ATC	Anaplastic thyroid cancer
BLCA	Bladder Urothelial Carcinoma
BRCA	Breast invasive carcinoma
BMSC	Bone marrow-derived mesenchymal stem cell
caRNA	Chromium associated RNA
ccRCC	Clear cell renal cell cancer
CESC	Cervical squamous cell carcinoma and endocervical adenocarcinoma
CHOL	Cholangiocarcinoma
COAD	Colon adenocarcinoma
CRC	Colorectal cancer
DFS	Disease-free survival
DLBC	Lymphoid Neoplasm Diffuse Large B-cell Lymphoma
DLBCL	Diffuse large B-cell lymphoma
ESCA	Esophageal cancer
ESCC	Esophageal Squamous Cell Carcinoma
GBM	Glioblastoma multiforme
GC	Gastric cancer
HCC	Hepatocellular carcinoma
HNSC	Head and Neck squamous cell carcinoma
KICH	Kidney Chromophobe
KIRC	Kidney renal clear cell carcinoma
KIRP	Kidney renal papillary cell carcinoma
LAML	Acute Myeloid Leukemia
LGG	Brain Lower Grade Glioma
LIHC	Liver hepatocellular carcinoma
LUAD	Lung adenocarcinoma
LUSC	Lung squamous cell carcinoma
MeRIP	Methylated RNA immunoprecipitation
MeRIP-seq	Methylated RNA immunoprecipitation-sequencing
MESO	Mesothelioma
m5C	5-Methylcytosine
NSCLC	Non-small-cell lung cancer
Nsun5-KO	Nsun5 knockout
OS	Overall survival
PAAD	Pancreatic adenocarcinoma
PBMCs	Peripheral blood mononuclear cells
PCPG	Pheochromocytoma and Paraganglioma
PDX	Patient derived tumor xenograft
PRAD	Prostate adenocarcinoma
READ	Rectum adenocarcinoma
RIP-seq	RNA immunoprecipitation-sequencing
SARC	Sarcoma
SKCM	Skin Cutaneous Melanoma
STAD	Stomach adenocarcinoma
TCGA	The Cancer Genome Atlas
TGCT	Testicular Germ Cell Tumors
THCA	Thyroid carcinoma
THYM	Thymoma
UCB	Urothelial carcinoma of the bladder
UCEC	Uterine Corpus Endometrial Carcinoma
UCS	Uterine Carcinosarcoma
UCSC	University of California Santa Cruz
UVM	Uveal Melanoma
