# Supplementary material for: The Role of NSUN Family Genes in m5C Methylation and Diseases
Source: Biomedicines. 2025 Nov 30;13(12):2951. doi: 10.3390/biomedicines13122951 (PMC12731187; doi:10.3390/biomedicines13122951)
Supplement: Supplementary file 1 [file biomedicines-13-02951-s001.zip › biomedicines-3980700-supplementary.pdf]

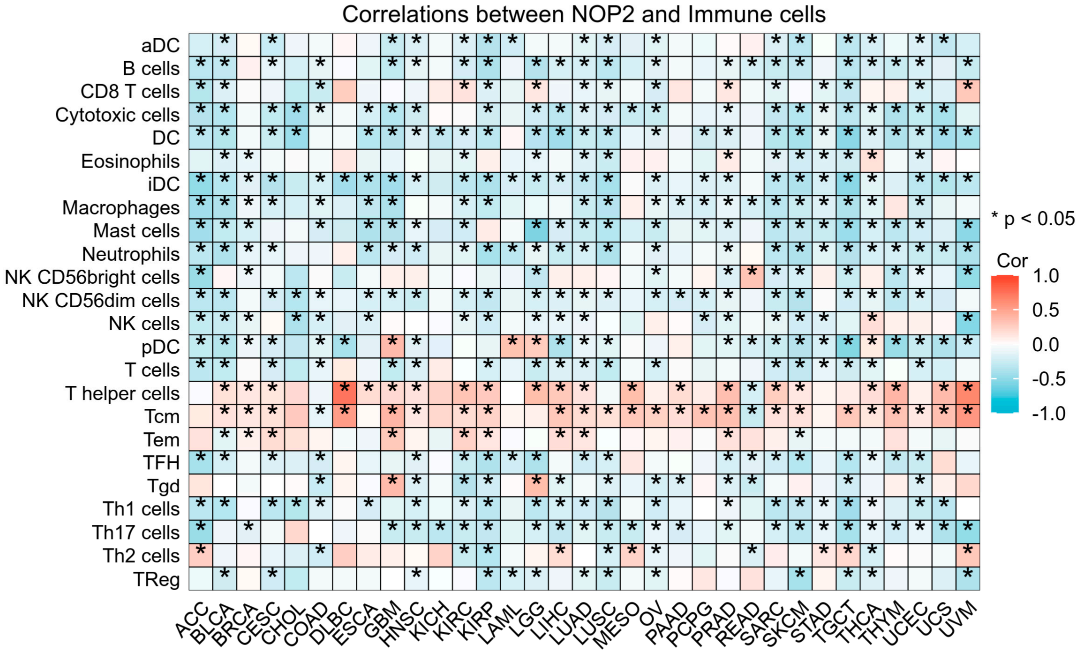

Figure S1. NOP2 expression correlation with immune infiltration.

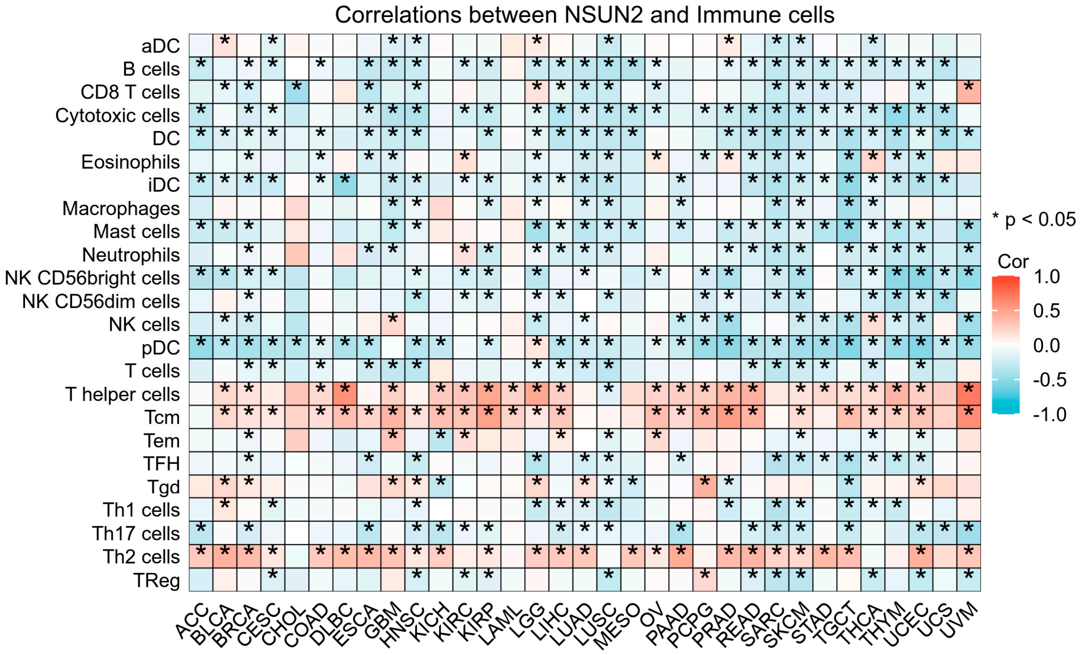

Figure S2. NSUN2 expression correlation with immune infiltration.

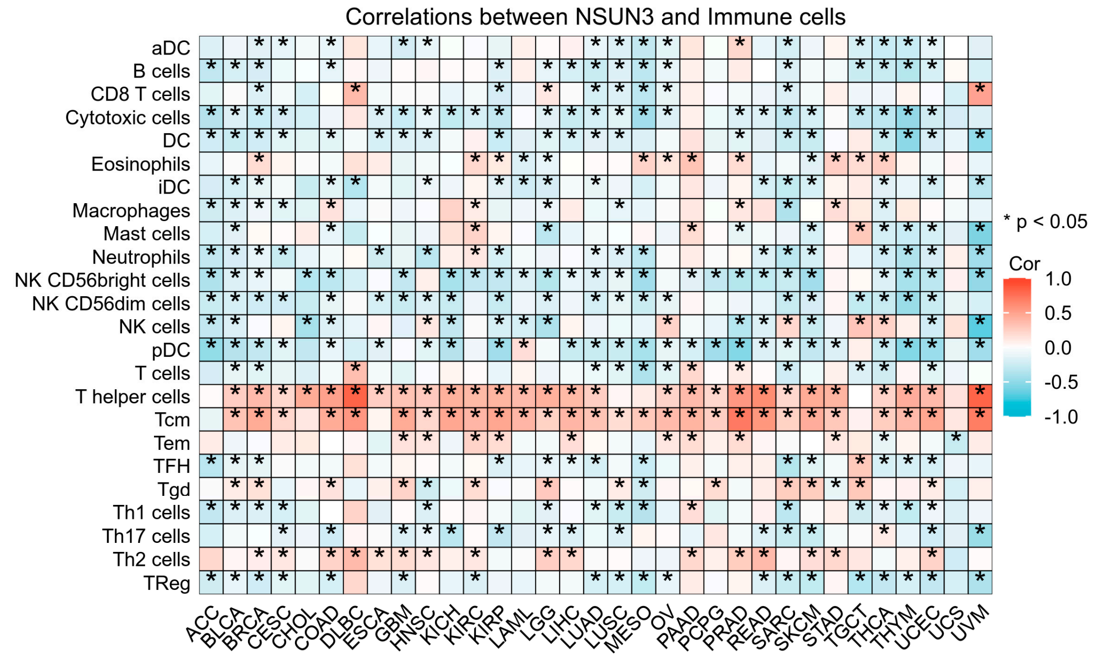

Figure S3. NSUN3 expression correlation with immune infiltration.

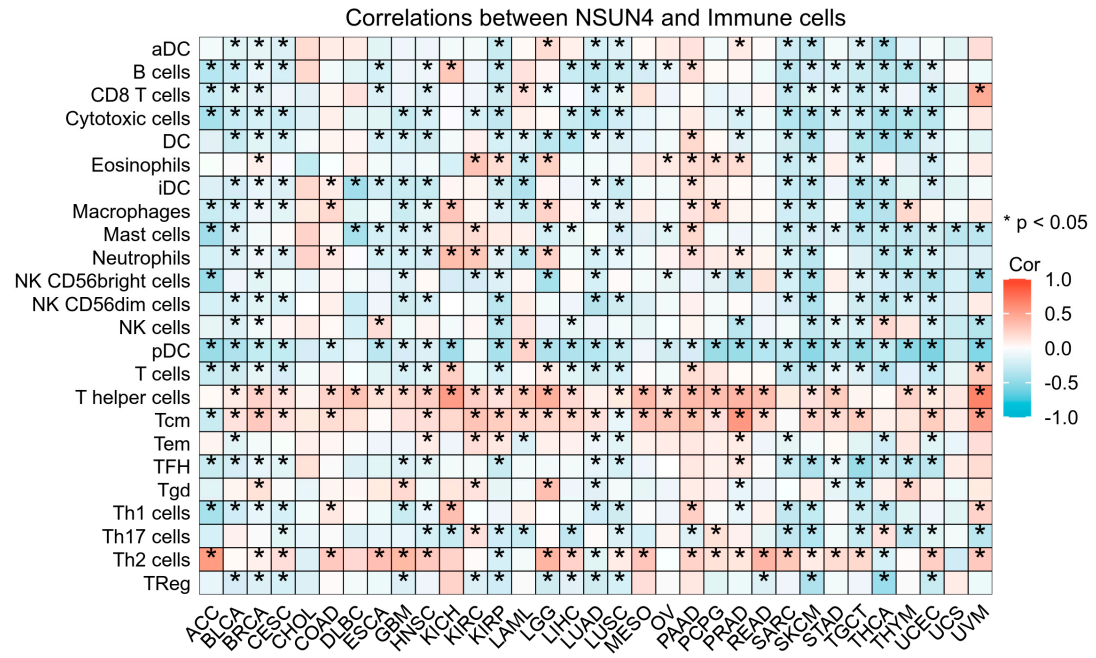

Figure S4. NSUN4 expression correlation with immune infiltration.

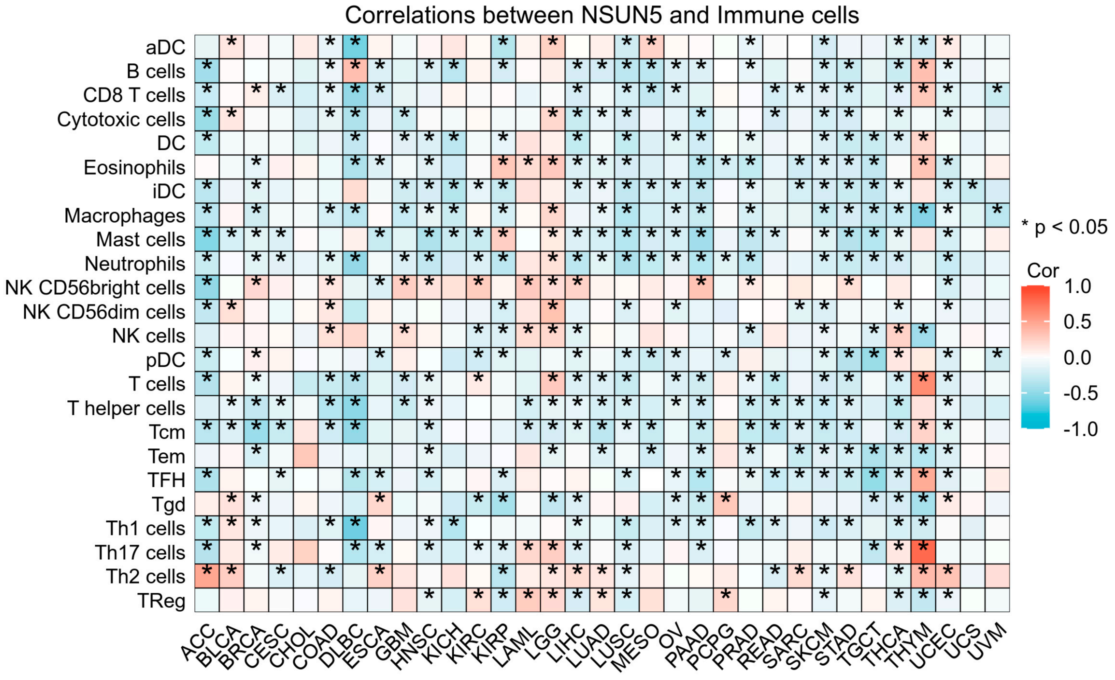

Figure S5. NSUN5 expression correlation with immune infiltration.

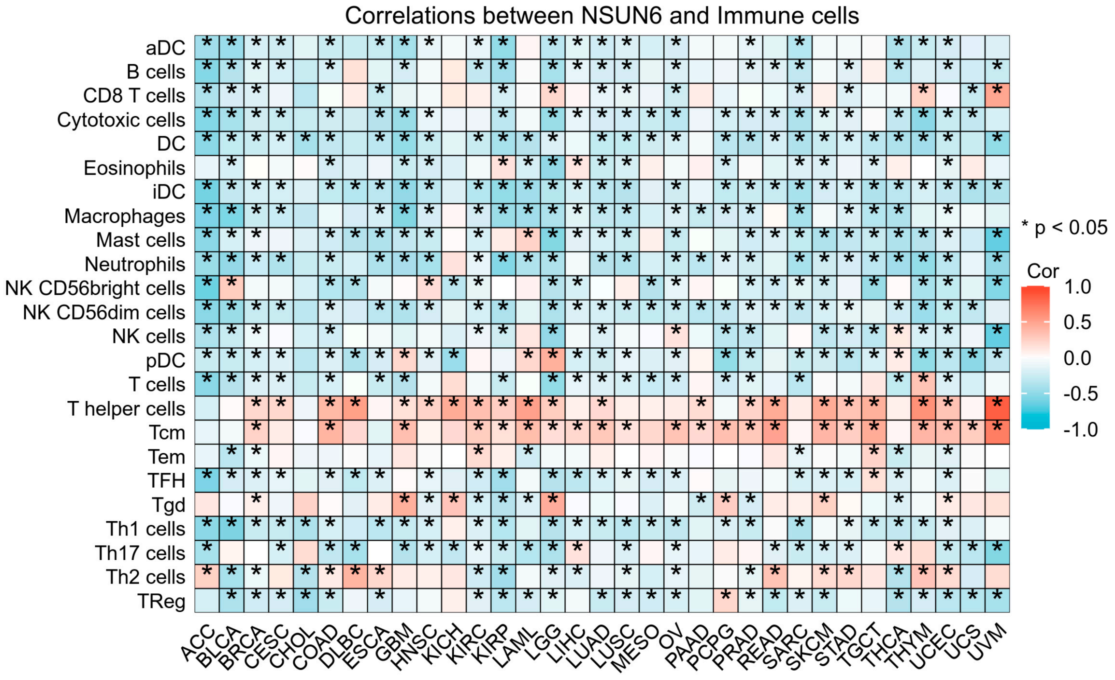

Figure S6. NSUN6 expression correlation with immune infiltration.

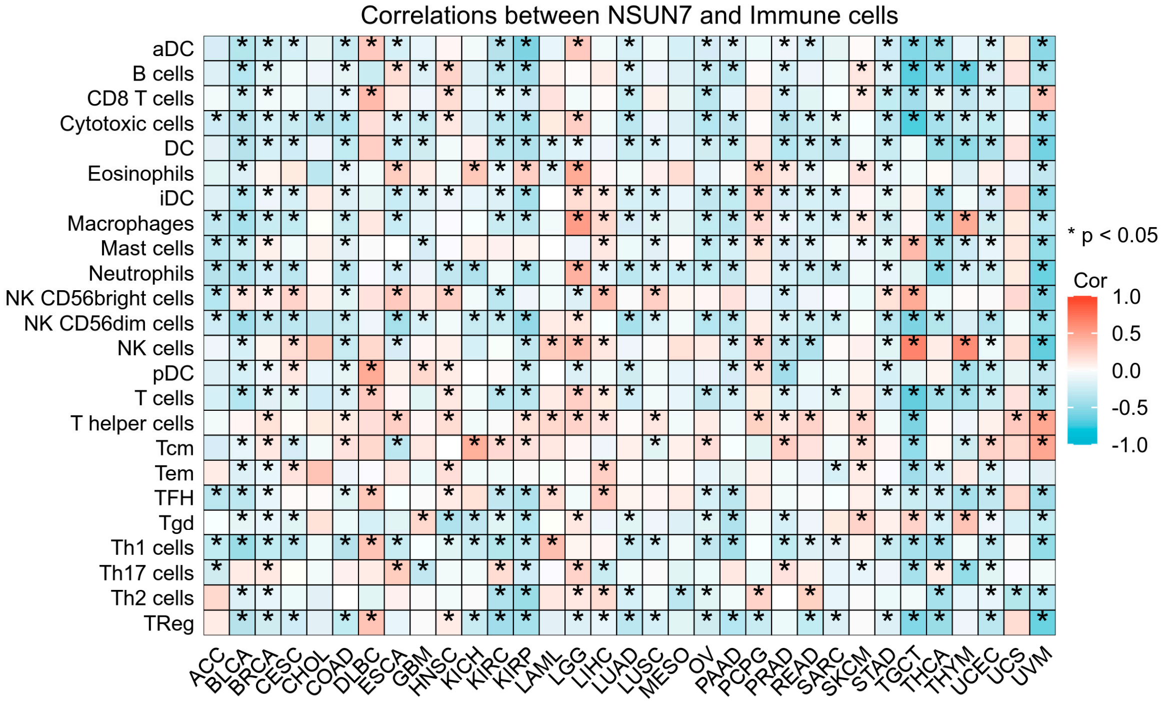

Figure S7. NSUN7 expression correlation with immune infiltration.
